# Identifying an Active Intermediate and Monitoring O─O Bond Formation in Water Oxidation by a Cobalt(III)‐TAML Complex

**DOI:** 10.1002/anie.202516165

**Published:** 2025-10-07

**Authors:** Deesha D. Malik, Yong‐Min Lee, Shunichi Fukuzumi, Kallol Ray, Wonwoo Nam

**Affiliations:** ^1^ Department of Chemistry and Nano Science Ewha Womans University Seoul 03760 South Korea; ^2^ College of Chemistry and Chemical Engineering Yan'an University, Yan'an Shaanxi Province 716000 P.R. China; ^3^ Department of Chemistry Humboldt‐Universität zu Berlin Brook‐Taylor‐Straße 2 Berlin 12489 Germany

**Keywords:** Catalytic and stoichiometric reactions, Cobalt TAML complex, O─O bond formation, Reaction mechanism, Water oxidation

## Abstract

A cobalt(III) −TAML complex, [(TAML)Co^III^]^−^ (TAML = tetraamido macrocyclic ligand), catalyzes water oxidation using one‐electron oxidants like cerium(IV) ammonium nitrate (CAN) or tris(4‐bromophenyl)ammonium hexachloroantimonate radical cation (TBPA^•+^). Two redox tautomers were characterized: a ligand‐centered Co(III)–OH species, [(TAML^•^⁺)Co^III^(OH)]^−^ (**1**), and a metal‐centered Co(IV)= O species, [(H‐TAML)Co^IV^(O)(HOTf)]^−^ (**2**), formed by oxidation with iodosylbenzene (PhIO) in the presence of triflic acid or with one‐electron oxidants. **1** readily reacts with water to evolve O_2_ via H_2_O_2_, while **2** remains unreactive under identical conditions. Electron‐transfer (ET) studies with electron donors such as diacetylferrocene (Ac_2_Fc) revealed that **1** is over 10⁴ times more reactive than **2**. The Marcus theory analysis showed a much lower reorganization energy (*λ*) for **1** (0.74 eV) compared to **2** (2.7 eV), explaining the dramatic rate difference. The detection of **1** during catalysis and kinetic isotope effect studies offers insight into O─O bond formation and underscores how redox‐site localization controls water oxidation reactivity.

## Graphical abstract

Two redox tautomers of a cobalt–TAML complex show strikingly different reactivity in water oxidation. The ligand‐centered Co(III)–OH species evolves O_2_ via H_2_O_2_, while the metal‐centered Co(IV) = O is inactive. Electron‐transfer kinetics and the Marcus analysis reveal how redox localization influences reactivity and O─O bond formation in high‐valent cobalt–oxo systems.

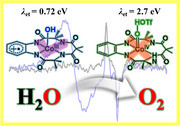

## Introduction

Inspired by the water oxidation at the oxygen evolving complex (OEC) composed of a Mn_4_CaO_5_ cluster in Photosystem II (PSII),^[^ ^1^, ^2^, ^3^, ^4^, ^5^, ^6^, ^7^, ^8^, ^9^, ^10^, ^11^, ^12^ ^]^ water oxidation by bioinspired metal catalysts has merited considerable attention. Accordingly, first‐row transition metal catalysts,^[^ ^13^, ^14^, ^15^, ^16^, ^17^, ^18^, ^19^, ^20^, ^21^, ^22^, ^23^ ^]^ as well as highly reactive second‐ and third‐row transition metal complexes, including the notable Ru‐based molecular syetems,^[^ ^24^, ^25^, ^26^, ^27^, ^28^, ^29^ ^]^ have been developed for catalytic water oxidation reactions over the past several decades. Although highly efficient catalysts have been reported in homogenous and heterogeneous catalytic water oxidation systems,^[^ ^13^, ^14^, ^15^, ^16^, ^17^, ^18^, ^19^, ^20^, ^21^, ^22^, ^23^, ^24^, ^25^, ^26^, ^27^, ^28^ ^]^ the nature of reactive intermediates and the O─O bond formation mechanisms still remain elusive. In most cases, high‐valent metal–oxo complexes, [(L)M*^n^* ^+^(O)]^(^ *^n−^* ^2)+^, have been implicated as reactive intermediates that form O─O bond to evolve O_2_ in the catalytic water oxidation reactions.^[^ ^29^, ^30^, ^31^, ^32^, ^33^, ^34^, ^35^, ^36^, ^37^, ^38^, ^39^, ^40^, ^41^, ^42^, ^43^, ^44^ ^]^ However, when redox‐active ligands (L) are employed, lower‐valent metal–oxo (or oxyl) complexes with oxidized ligands, such as [(L^•+^)M^(^ *^n−^* ^1)^^+^(O)]^(^ *^n−^* ^2)+^ and [(L^2+^)M^(^ *^n−^* ^2)+^(O)]^(^ *^n−^* ^2)+^, can also act as potential reactive intermediates.^[^ ^38^, ^45^, ^46^, ^47^, ^48^, ^49^, ^50^, ^51^, ^52^, ^53^, ^54^, ^55^, ^56^, ^57^, ^58^, ^59^, ^60^, ^61^, ^62^ ^]^ Therefore, identification of the nature of the active species, such as high‐valent metal–oxo or lower‐valent metal oxo (or oxyl) complexes with oxidized ligands, is worth being investigated intensively.

Cobalt complexes bearing redox‐innocent TAML ligands (TAML = tetraamido macrocyclic tetra‐anionic ligand), [(TAML)Co^III^]*^−^*, have been reported as homogeneous catalysts in a number of electrocatalytic water oxidation reactions.^[^ ^45^, ^46^, ^52^ ^]^ Gupta and co‐workers reported the electrochemical water oxidation by a biuret‐modified TAML cobalt complex, [(b‐TAML)Co^III^]^–^, at basic pHs, leading to the O_2_ evolution via the formation of a formal Co(V)‐oxo species as an active oxidant.^[^ ^45^ ^]^ Recently, Zhang and co‐workers used modified [(TAML)Co^III^]^−^ catalysts in electrocatalytic water oxidation in aqueous solution.^[^ ^46^ ^]^ Based on the experimental and DFT results, phosphate‐assisted water nucleophilic attack to [(TAML)Co^IV^(O)] was proposed in the O─O bond formation process.^[^ ^46^ ^]^ Very recently, Zhang, Shao, and co‐workers captured two key intermediates, [(TAML)Co^III^(OH)]^2−^ and [(TAML)Co^III^(OOH)]^2−^, and proposed their structures from ^18^O‐labeling and collision‐induced dissociation studies.^[^ ^52^ ^]^ More recently, two redox tautomers of metal–oxo/hydroxo complexes, such as a cobalt(III)–hydroxo complex with a one‐electron oxidized ligand, [(TAML^•+^)Co^III^(OH)]^−^ (**1**), and a cobalt(IV)–oxo complex with a protonated TAML ligand binding triflic acid (HOTf), [(H‐TAML)Co^IV^(O)(HOTf)]^−^ (**2**), were produced and characterized spectroscopically by some of us; these redox tautomers were found to be in a thermally slow equilibrium.^[^ 5^7^ ^]^

In the present study, we investigate in detail the possible involvement of **1** and **2** in O─O bond formation reactions. Consistent with the previous reports, we find that [(TAML)Co^III^]^−^ acts as an efficient catalyst for water oxidation in the presence of one‐electron oxidants, such as cerium(IV) ammonium nitrate (CAN)^[^ ^63^ ^]^ and tris(4‐bromophenyl)ammonium hexachloroantimonate radical cation (TBPA^•+^),^[^ ^64^ ^]^ with a high turnover number (TON). More importantly, we demonstrate that the Co^III^(OH) moiety in **1** is an active oxidant that reacts with water to evolve O_2_. In contrast, the Co^IV^(O) complex **2**, which is in slow equilibrium with **1**, surprisingly does not react with water under identical reaction conditions. We also report the electron‐transfer (ET) reactions of **1** and **2** with electron donors, such as acetylferrocene (AcFc), affording the ET reorganization energies (*λ*) of 0.74 and 2.7 eV for **1** and **2**, respectively.^[^ ^65^, ^66^ ^]^ This large difference in the *λ* values of **1** and **2** provides valuable insights into the water oxidation mechanism in which **1**, but not **2**, is an active intermediate that reacts with water to evolve O_2_.

## Results and Discussion

### Catalytic Water Oxidation

**Figure 1:**
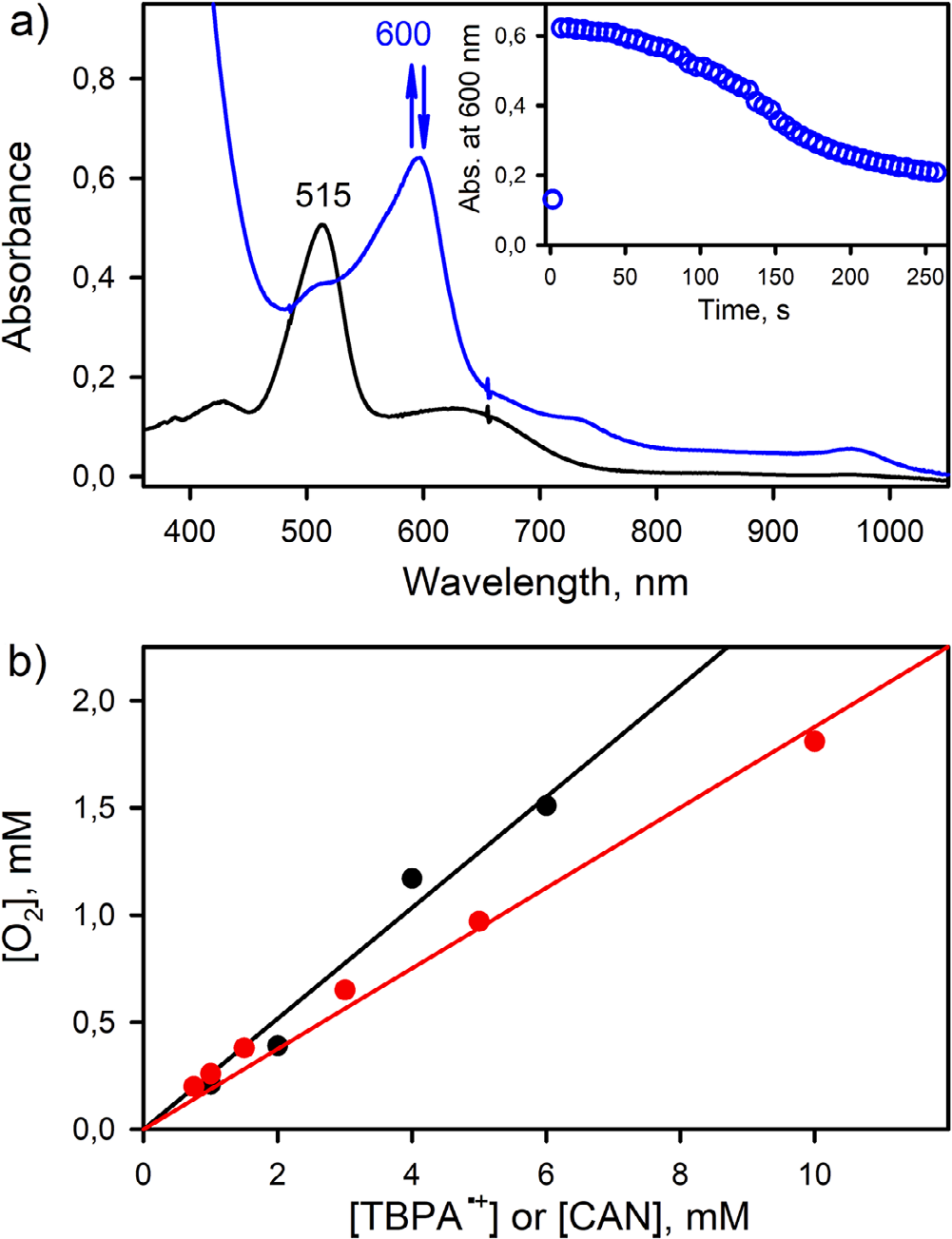
a) UV–vis spectra recorded during the water oxidation by **1** in Ar‐saturated acetone containing Li[Co^III^(TAML)]•3H_2_O (0.10 mM, black line), CAN (1.0 mM), and water (0.50 M) at 25 °C. Inset shows time trace monitored at 600 nm due to **1** (blue circles). b) Plots of concentrations of CAN (red circles) and TBPA^•+^ (black circles) versus concentrations of O_2_ produced in the catalytic water oxidation using Li[Co^III^(TAML)]•3H_2_O (0.10 mM), CAN (0.75–10 mM), or TBPA^•+^ (1.0–6.0 mM), and water (0.50 M) in Ar‐saturated acetone at 25 °C (see Tables S1 and S2).

**Figure 2:**
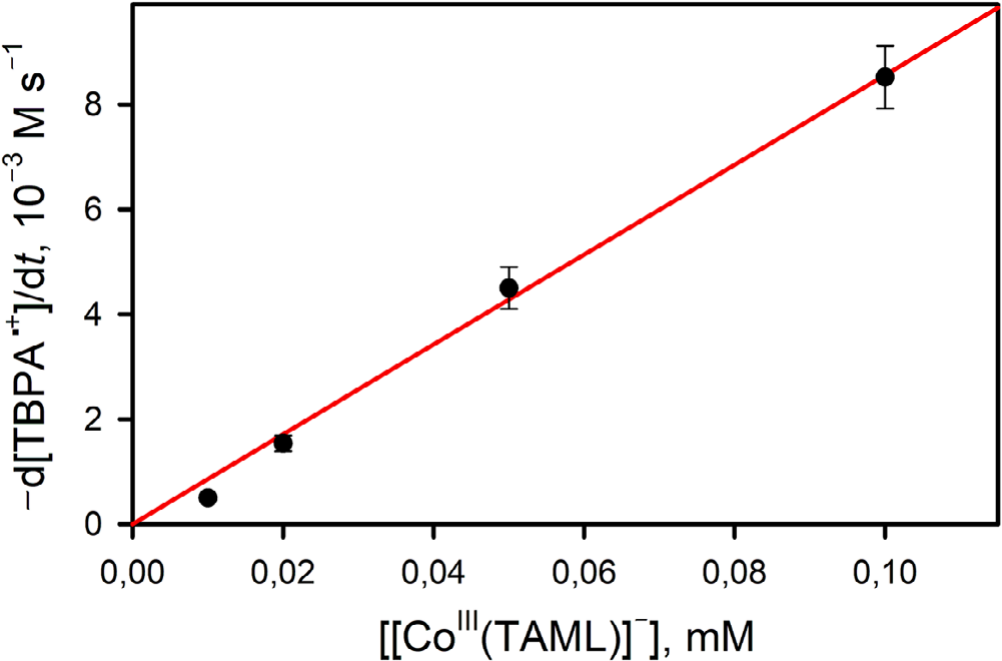
Plot of the concentrations of [(TAML)Co^III^]^−^ versus initial decay rates of TBPA^•+^ obtained in the catalytic water oxidation reaction by Li[Co^III^(TAML)]•3H_2_O (0.010–0.10 mM) in the presence of TBPA^•+^ (0.50 mM) and H_2_O (0.50 M) in Ar‐saturated acetone at 25 °C.

**Figure 3:**
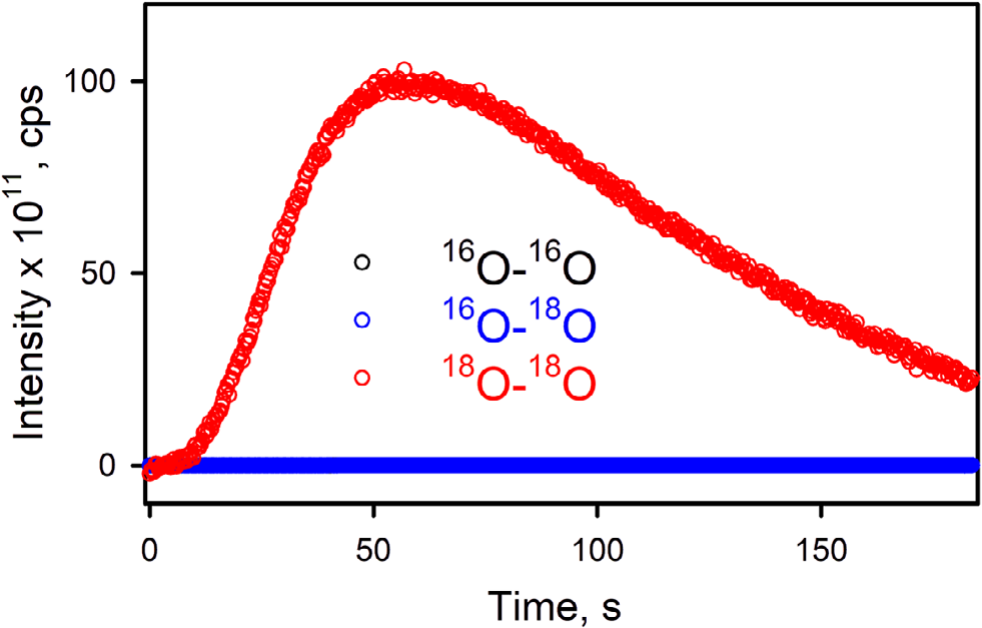
Time profile of mass spectra monitoring O_2_ isotopes [32 (^16^O–^16^O, black circles), 34 (^16^O–^18^O, blue circles), and 36 (^18^O–^18^O, red circles)] produced in the catalytic water oxidation by Li[Co^III^(TAML)]•3H_2_O (0.050 mM), TBPA^•+^ (5.0 mM), and H_2_^18^O (1.0 M) in N_2_‐saturated acetone at 25 °C. The amounts of ^16^O–^16^O (black circles) and ^16^O–^18^O (blue circles) produced were negligible.

We examined the catalytic activity of [(TAML)Co^III^]^−^ in the oxidation of water by one‐electron oxidants, such as CAN and TBPA^•+^. After the catalytic reaction, [(TAML)Co^III^]^−^ remained intact. When CAN (or TBPA^•+^) was added to an Ar‐saturated acetone solution containing a catalytic amount of [(TAML)Co^III^]^−^, the color of the reaction solution was immediately changed from purple to blue; the electronic absorption band at 515 nm corresponding to the starting [(TAML)Co^III^]^−^ complex disappeared with the formation of a new band at 600 nm corresponding to the intermediate **1** (vide infra), as shown in Figures 1a and S1.^[^ ^57^, ^58^, ^59^ ^]^ The oxidized product of water was O_2_, which was analyzed by gas chromatography (GC). In the catalytic oxidation of water by [(TAML)Co^III^]^−^ and CAN (or TBPA^•+^), O_2_ was evolved with the yield of 24 ± 3% according to Equations (1) and (2), and turnover numbers (TON = mol of O_2_ per mol of catalyst) of 30 and 18 were obtained in the reactions of TBPA^•+^ and CAN, respectively (Figure 1b; Tables S1 and S2). The TON increased to 66 by decreasing the amount of the cobalt catalyst to 5.0 µM in the presence of CAN (Table S3). When the catalyst concentration decreased from 0.10 mM to 5.0 µM in the reaction of TBPA^•+^, the TON increased from 14 to 150 (Table S4). Further, the initial rate of the catalytic water oxidation by TBPA^•+^ with [(TAML)Co^III^]^−^ increased linearly with increasing concentration of [(TAML)Co^III^]^−^ (Figure 2 and Table S5). Furthermore, when the catalytic water oxidation by [(TAML)Co^III^]^−^ and TBPA^•+^ was performed with ^18^O‐labeled water (H_2_^18^O), ^18^O_2_ was produced as the sole product, as detected by gas chromatography‐mass spectrometry (GC‐MS) (Figure 3). The latter result supports that the source of oxygen in the O_2_ product was H_2_O. No O_2_‐evolution was observed in the absence of [(TAML)Co^III^]^−^ and the one‐electron oxidants, such as TBPA^•+^ and CAN, under the reaction conditions.

(1) $$4\text{TBP}A^{•+}+24H_{2}O\overset{{\left[\left(\text{TAML}\right){\mathrm{Co}}^{\text{III}}\right]}^{-}}{\to }\text{TBPA}+O_{2}+4H^{+}$$

(2)

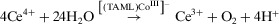




### Water Oxidation by Redox Tautomers, **1** and **2**

**Figure 4:**
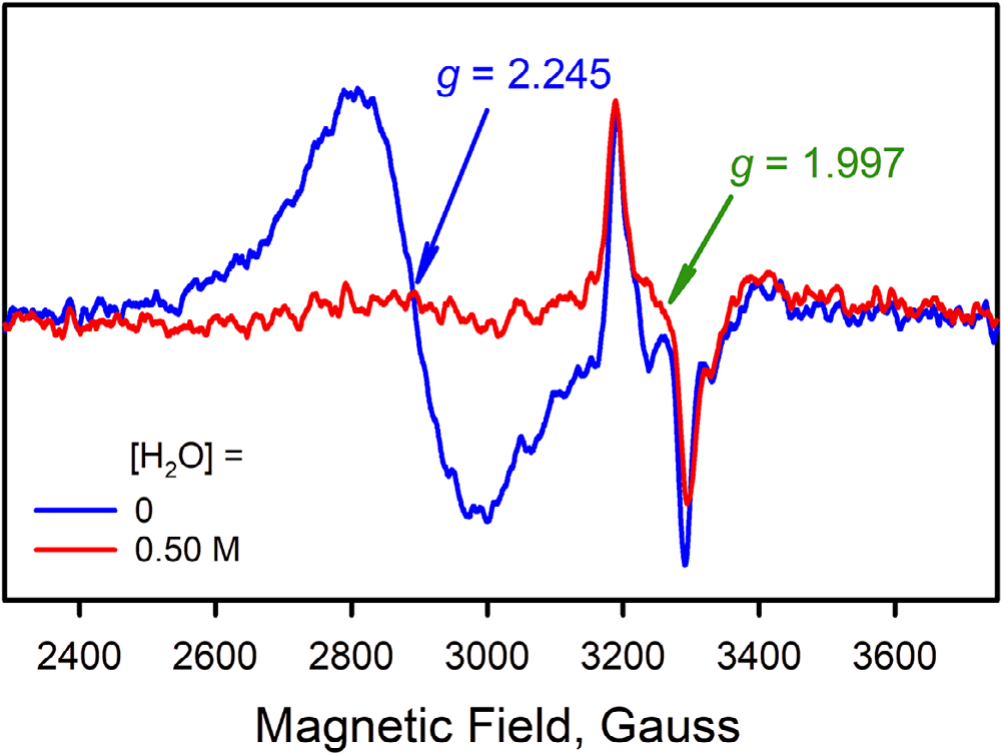
Solution EPR spectra of the intermediates **1** and **2** before (blue line) and after (red line) the addition of H_2_O (0.50 M) in Ar‐saturated acetone at −40 °C. Intermediates **1** and **2** were generated by reacting Li[Co(TAML)]•3H_2_O (2.0 mM) with PhIO (3.0 equiv.) and HOTf (5.0 equiv.) in Ar‐saturated acetone at −40 °C. It is noted that the reaction of **1** with H_2_O is much faster than the conversion of **2** to **1**.

In our effort to determine the nature of the reactive intermediate involved in [(TAML)Co^III^]^−^ catalyzed water oxidation reaction, we investigated the plausible mediation of **1** and **2** in the O─O bond formation step. For this purpose, **1** and **2** were generated by reacting [(TAML)Co^III^]^−^ with PhIO in the presence of HOTf in Ar‐saturated acetone (Figure S2a) as reported previously.^[^ ^57^ ^]^ **1** could also be generated by the oxidation of [(TAML)Co^III^]^−^ with one equiv. of CAN or TBPA^•+^ in Ar‐saturated acetone at 25 °C (Figure S2b).^[^ ^57^, ^58^, ^59^ ^]^ Both the intermediates **1** and **2** are EPR active in solution at −40 °C, as shown previously (Figure 4, blue trace);^[^ ^57^ ^]^ a broad EPR signal at *g* = 2.245 is an average rhombic signal of **1**, and another EPR signal at *g* = 1.997 agrees with the average anisotropic signal of **2** (Figure 4).^[^ ^57^, ^67^ ^]^ Upon addition of water (0.50 M) to an Ar‐saturated acetone solution containing a mixture of **1** and **2** at −40 °C, the EPR signal at *g* = 2.245 due to **1** disappeared, whereas the EPR signal at *g* = 1.997 due to **2** remained intact, as shown in Figure 4. As the equilibrium between **1** and **2** is previously reported to be slow,^[^ ^57^ ^]^ this result suggests that H_2_O performs a faster ligand‐centered reduction of the [(TAML^•+^)Co^III^(OH)]^−^ moiety in **1** than the metal‐centered reduction of [(TAML‐H)Co^IV^(O)(HOTf)]^−^ in **2** (vide infra).^[^ ^53^, ^54^, ^55^ ^]^ See also the ET reaction section for the discussion of the reactivity difference of **1** and **2**.

### Stoichiometric Water Oxidation by **1**

**Figure 5:**
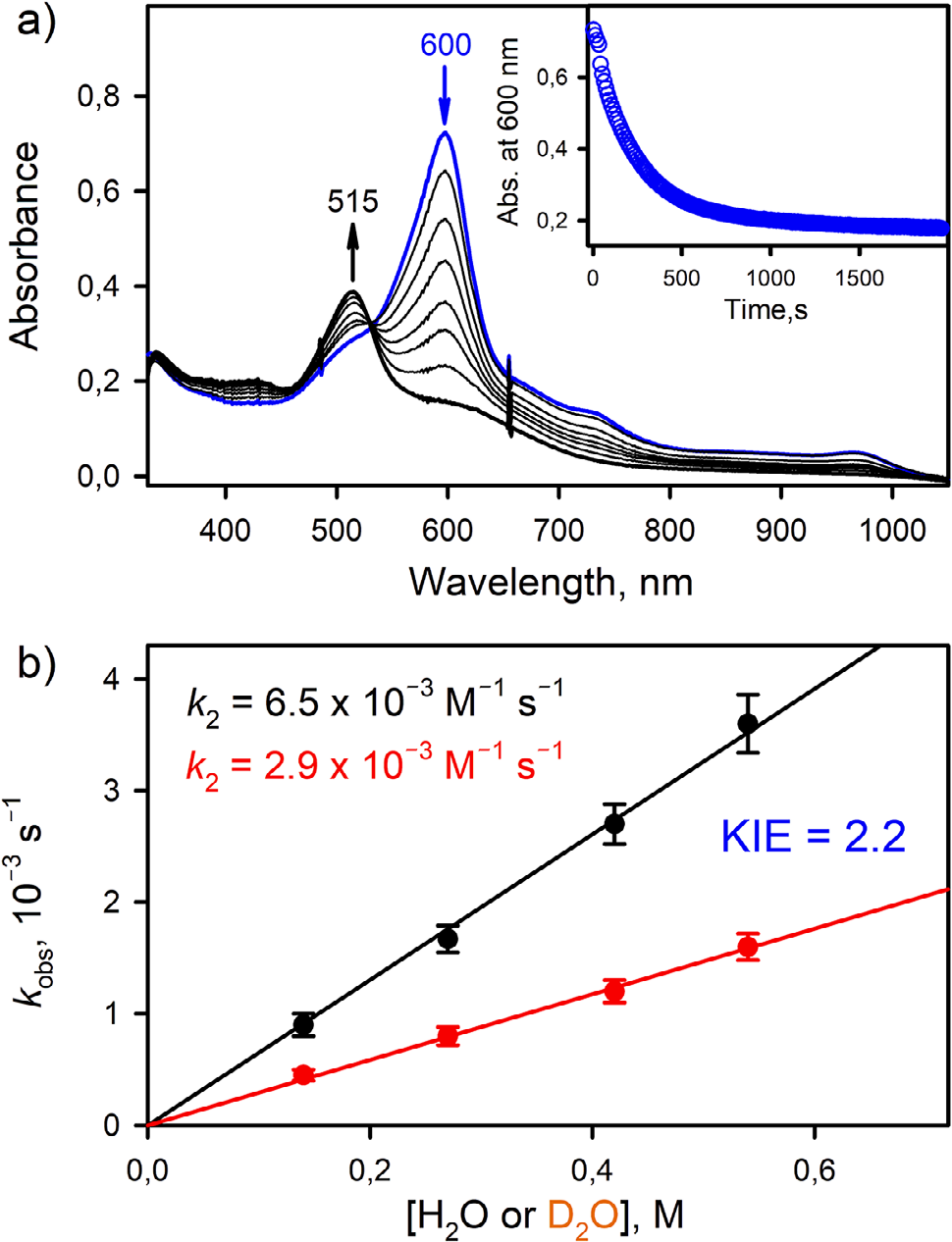
a) UV–vis spectral changes showing the decay of **1** (0.10 mM, blue line) to [(TAML)Co^III^]^−^ (black line) in the presence of H_2_O (0.50 M) in Ar‐saturated acetone at 25 °C. Inset shows time trace monitored at 600 nm due to **1**. b) Plots of pseudo‐first‐order rate constants versus concentrations of H_2_O (black circles) and D_2_O (red circles) in water oxidation by **1** in Ar‐saturated acetone at 25 °C. Intermediate **1** was generated by reacting Li[Co(TAML)]•3H_2_O (0.10 mM) with PhIO (0.50 equiv.) in the presence of HOTf (0.50 equiv.) in Ar‐saturated acetone at 25 °C.

Addition of water to an acetone solution of **1**, which was generated by reacting [(TAML)Co^III^]^−^ with 0.50 equiv. of PhIO in the presence of 0.50 equiv. of HOTf in Ar‐saturated acetone at 25 °C, resulted in a sharp color change from blue to pink, with the UV–vis spectral change showing the decay of **1** to the starting [(TAML)Co^III^]^−^ complex (Figure 5a). The decay of **1** obeyed the first‐order kinetics, and the decay rate was proportional to the amount of H_2_O added, leading us to determine the second‐order rate constant (*k*_2_) of 6.5 × 10^−3^ M^−1^ s^−1^ at 25 °C. We also determined the *k*_2_ value of 2.9 × 10^−3^ M^−1^ s^−1^ with D_2_O, thereby obtaining a deuterium kinetic isotope effect (KIE) value of 2.2 (Figure 5b). The KIE of 2.2 indicates that the rate‐determining step in the water oxidation by **1** involves the O─H bond cleavage of H_2_O.^[^ ^56^, ^68^, ^69^ ^]^ **1** produced by the oxidation of [(TAML)Co^III^]^−^ with CAN and TBPA^•+^ could also oxidize water to afford the deuterium kinetic isotope effects (KIEs) of 1.4 and 1.6, respectively (Figures S3 and S4). After the reaction was completed, the inorganic product was found to be Co^III^ species, which was analyzed by electrospray ionization mass spectrometer (ESI‐MS) (Figure S5) and EPR spectroscopy (Figure S6).

As shown in Figures S7 and S8 and Tables S6 and S7, the amount of O_2_ produced increased proportionally with the amount of **1** used in the reaction. When 1 equiv. of CAN (Figure S7 and Table S6) or 0.50 equiv. of PhIO and 0.50 equiv. of HOTf (Figure S8 and Table S7) was used, 25% ± 3% (based on **1**) yield of O_2_ was obtained. Thus, the stoichiometry of the water oxidation by **1** is shown in Equation (3); 4 equiv. of **1** reacts with one molecule of water to produce one molecule O_2_. [(TAML)Co^III^(OH)]^2−^ in Equation (3) may further react with H^+^ to yield [(TAML)Co^III^]^−^ and H_2_O.

(3) $$\begin{array}{cc} & 4{\left[\left(\mathrm{TAM}L^{•+}\right)Co^{\mathrm{III}}\left(\mathrm{OH}\right)\right]}^{-}+H_{2}O\\ & \;\to {\left[\left(\mathrm{TAML}\right)Co^{\mathrm{III}}\right]}^{-}+O_{2}+3H^{+}\\ & \;+3{\left[\left(\mathrm{TAML}\right)Co^{\mathrm{III}}\left(\mathrm{OH}\right)\right]}^{2-} \end{array}$$

### Detection of a Cobalt‐Peroxo Intermediate in the Stoichiometric Water Oxidation by **1**

We have recently reported the cold‐spray ionization time‐of‐flight mass spectrometry (CSI‐MS) data for the intermediate **1** in Ar‐saturated acetone, showing peaks with the mass‐to‐charge (*m/z*) ratios of 446.1 and 448.1 corresponding to [(TAML)Co(^16^OH)]^+^ and ^18^O‐labeled species [(TAML)Co(^18^OH)]^+^, respectively.^[^ ^57^ ^]^ Interestingly, in the present study, upon addition of water to **1** at −40 °C in Ar‐saturated acetone, a peak at *m/z* of 461.1 corresponding to a cobalt‐peroxo complex, [(TAML)Co(OO)]^−^, was observed in negative mode (Figure S9). When **1**–^16^O was reacted with H_2_^18^O we observed a two‐mass unit shift from 461.1 to 463.1 corresponding to [(TAML)Co(^16^O^18^O)]^−^ and a four‐mass unit shift from 461.1 to 465.1 corresponding to [(TAML)Co(^18^O^18^O)]^−^. The observation of the mixed ^16^O and ^18^O in the cobalt‐peroxo complex demonstrates unambiguously that **1**–^16^O, which was generated with PhI^16^O, reacted with H_2_^18^O to produce [(TAML)Co(^16^O^18^O)]^−^. The peak at *m/z* of 465.1 corresponding to [(TAML)Co(^18^O^18^O)]^−^ the product of the reaction of **1**–^18^O and H_2_^18^O; **1**–^18^O was presumably generated from the ^18^O‐exchange between **1**–^16^O and H_2_^18^O and the degree of the ^18^O‐incorporation from H_2_^18^O into the cobalt‐peroxo depends on the relative rate of the ^18^O‐exchange between **1**–^16^O and H_2_^18^O.^[^ ^57^, ^59^ ^]^

### Reaction of **1** with H_2_O_2_ to Produce O_2_

**Figure 6:**
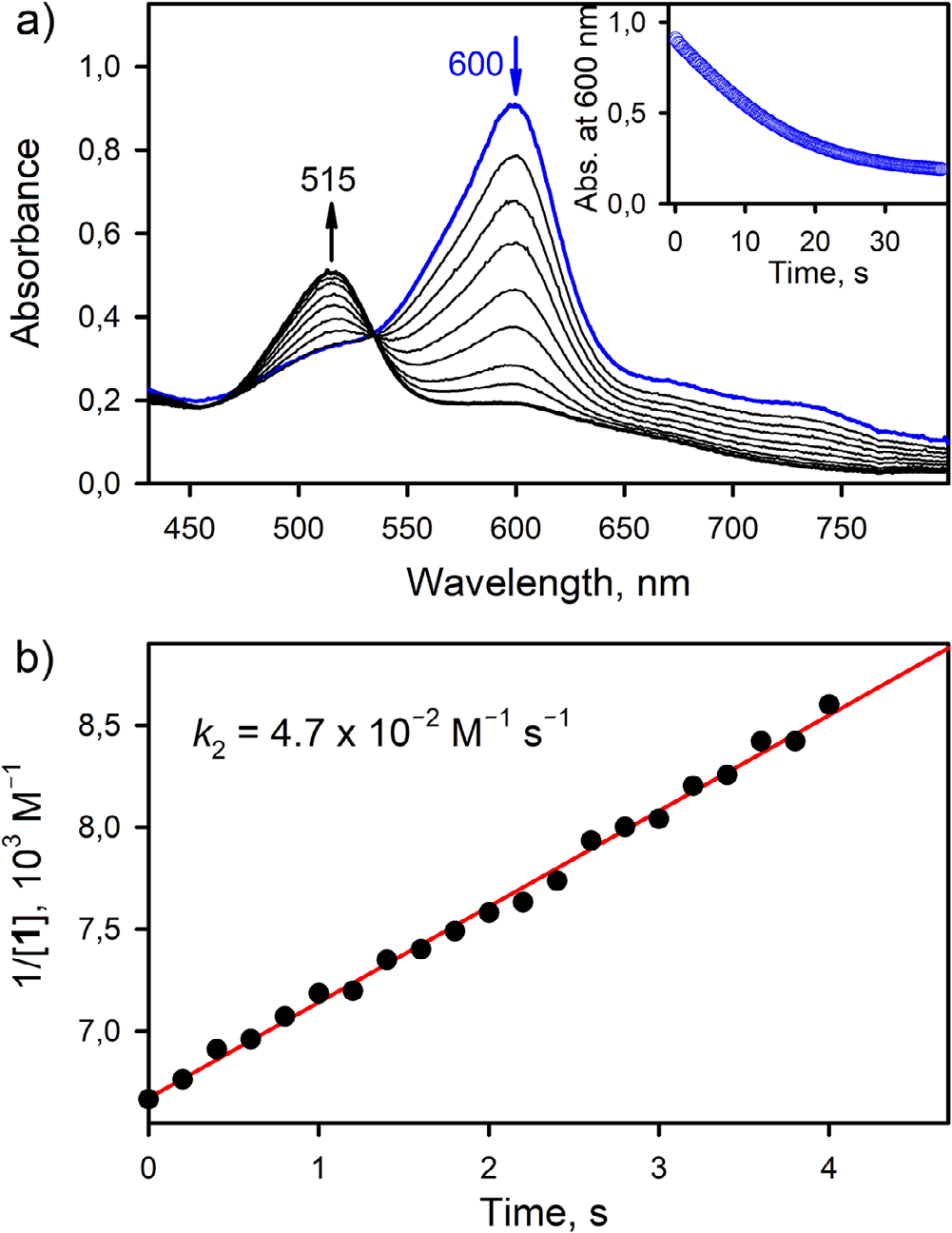
a) UV–vis spectral changes showing the decay of **1** (0.15 mM) in the presence of stoichiometric amount of H_2_O_2_ (0.075 mM, diluted from a 30% H_2_O_2_ aqueous solution) in Ar‐saturated acetone at 25 °C. Inset shows time trace monitored at 600 nm due to **1**. See Equation (4) for the 2:1 ratio of H_2_O_2_ and **1**. b) A second‐order plot of 1/[**1**] versus time in H_2_O_2_ oxidation by **1** in Ar‐saturated acetone at 25 °C. **1** was generated by oxidizing Li[Co(TAML)]•3H_2_O (0.15 mM) with CAN (1.0 equiv.) in Ar‐saturated acetone at 25 °C.

We also found that **1** reacted rapidly with 0.50 equiv. of H_2_O_2_ to produce O_2_ (Figures 6a and S10 and Table S8). The stoichiometry of the reaction of **1** with H_2_O_2_ is given by Equation (4),

(4) $$\begin{array}{cc} & 2{\left[\left(\mathrm{TAM}L^{•+}\right)Co^{\mathrm{III}}\left(\mathrm{OH}\right)\right]}^{-}+H_{2}O_{2}\\ & \;\to 2{\left[\left(\mathrm{TAML}\right)Co^{\mathrm{III}}\left(\mathrm{OH}\right)\right]}^{2-}+O_{2}+2H^{+} \end{array}$$

where [(TAML)Co^III^(OH)]^2−^ further reacts with H^+^ to produce [(TAML)Co^III^]^−^ and H_2_O. The reaction of H_2_O_2_ with **1** at 25 °C was too fast to be monitored by a conventional UV–vis spectrophotometer and was therefore analyzed using stopped‐flow techniques. A second‐order plot of 1/[(TAML^•+^)Co^III^(OH)]^−^ (1/[**1**]) versus time afforded *k*_2_ of 4.7 × 10^2^ M^−1^ s^−1^ in Ar‐saturated acetone at 25 °C (Figure 6b), which is 7.2 x 10^4^ times greater than the rate of the reaction of **1** with H_2_O. The latter result suggests that H_2_O_2_ can be produced in the reaction of **1** and H_2_O if a large concentration of H_2_O (i.e., >7.2 x 10^4^ times larger than that of H_2_O_2_) is used. Indeed, 40% H_2_O_2_ was produced as the main product in the reaction of **1** and H_2_O when the concentration of H_2_O (e.g., 15 M) is 1.5 x 10^5^ 150 000 times larger than that of **1** (e.g., 0.10 mM), as shown in Figure S11 (the maximum yield based on Equation (5) is 50%; Figure S12 and Table S9). The amount of H_2_O_2_ produced was determined by the spectroscopic titration with oxo[5,10,15,20‐tetra(4‐pyridyl)porphyrinato]titanium(IV) (Ti‐TPyP) (see Experimental Section);^[^ ^70^ ^]^ the selectivity for the two‐electron oxidation of H_2_O to H_2_O_2_ was 88%. The selectivity for the formation of H_2_O_2_ decreased with increasing concentration of **1** (i.e., 64% at 0.15 mM) and decreasing concentration of H_2_O (i.e., 24% at 5 M) (Table S10).

(5) $$\begin{array}{cc} & 2{\left[\left(\mathrm{TAM}L^{•+}\right)Co^{\mathrm{III}}\left(\mathrm{OH}\right)\right]}^{-}+H_{2}O\\ & \;\to H_{2}O_{2}+{\left[\left(\mathrm{TAML}\right)Co^{\mathrm{III}}\right]}^{-}+H^{+}\\ & \;+{\left[\left(\mathrm{TAML}\right)Co^{\mathrm{III}}\left(\mathrm{OH}\right)\right]}^{2-} \end{array}$$

**Scheme 1:**
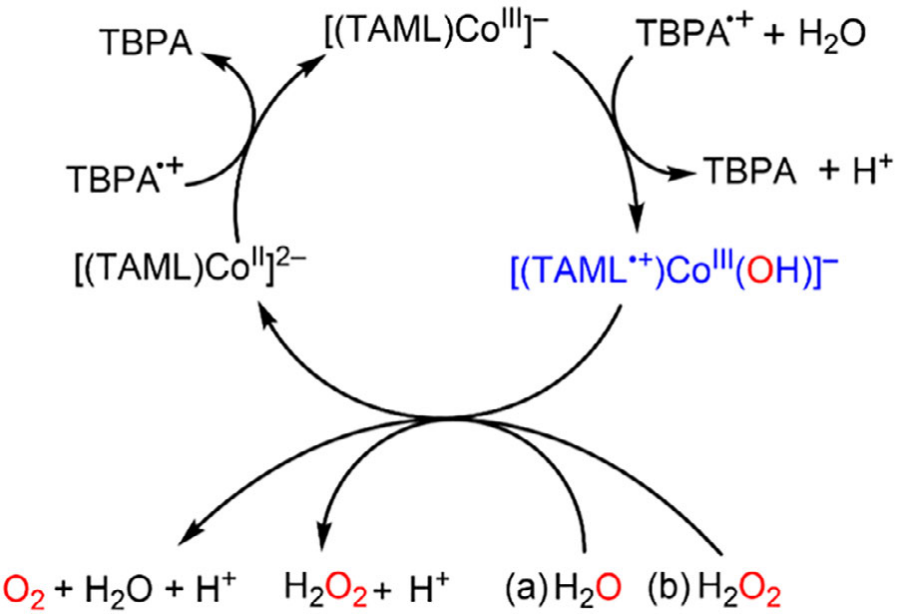
Catalytic cycle for the a) H_2_O and b) H_2_O_2_ oxidation by TBPA^•+^ with [(TAML)Co^III^]^−^

The stoichiometry of the two‐electron oxidation of H_2_O by **1** to produce H_2_O_2_ is given by Equation (5), where [(TAML)Co^III^(OH)]^2−^ may further react with H^+^ to produce [(TAML)Co^III^]^−^ and H_2_O. It was confirmed that [(TAML)Co^III^]^−^ was stable in the presence of H_2_O_2_ in Ar‐saturated acetone (Figure S13).^[^ ^71^ ^]^ The detection of the H_2_O_2_ in the water oxidation by **1** indicates that the four‐electron oxidation of H_2_O by **1** (Equation (3)) proceeds via two steps, such as the two‐electron oxidation of H_2_O to H_2_O_2_ (Equation (5); Scheme 1, reaction *a*), followed by a rapid two‐electron oxidation of H_2_O_2_ by **1** to evolve O_2_ (Equation (4); Scheme 1, reaction *b*). [(TAML)Co^III^(OH)]^2−^ in Equation (5) may further react with H^+^ to yield [(TAML)Co^III^]^−^ and H_2_O.

**Scheme 2:**
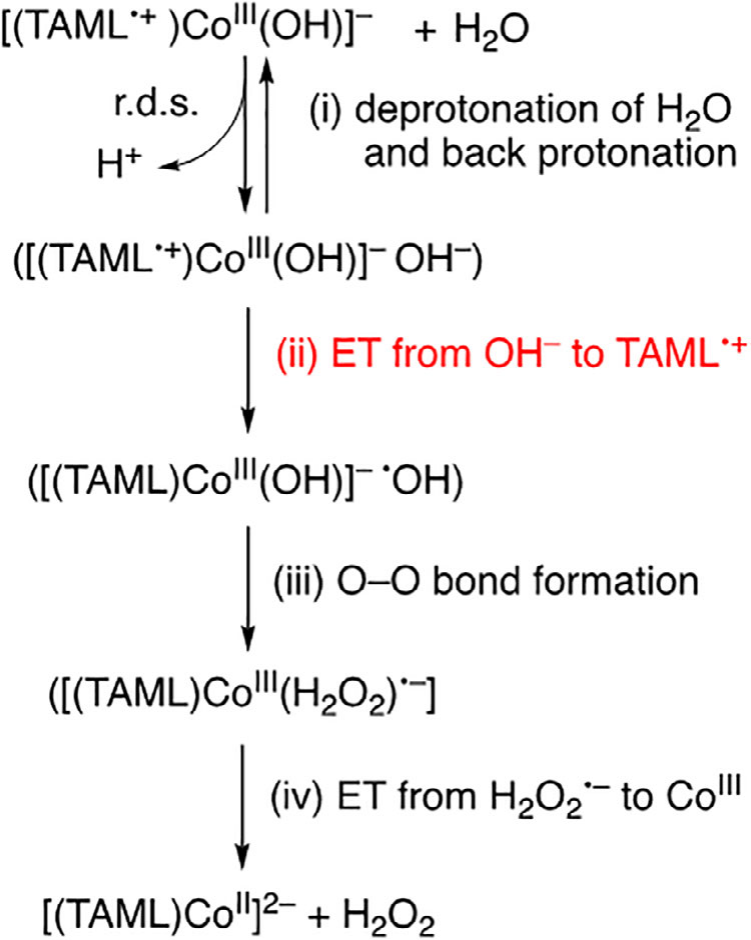
Proposed mechanism for water oxidation by [(TAML^•+^)Co^III^(OH)]^−^ **(1)**.

The reason why **1** reacts with H_2_O but **2** shows no reactivity toward H_2_O can be explained by clarifying the mechanism of the reaction of **1** with H_2_O. The common mechanism, such as the nucleophilic attack of H_2_O to the high‐valent metal–oxo species, cannot be applied to the case of **1,** that is, the Co(III)–hydroxo species with the TAML^•+^ ligand.^[^ ^72^ ^]^ Because the TAML^•+^ ligand in **1** is reduced to TAML by the reaction with H_2_O, the electron‐transfer reduction of TAML^•+^ is involved in the mechanism of the reaction of **1** with H_2_O. A proposed mechanism of water oxidation by **1** is shown in Scheme 2, where (i) the deprotonation of H_2_O is followed by electron transfer from OH^−^ (*E*_ox_ versus SCE = 0.55 V)^[^ ^73^ ^]^ to the TAML^•+^ moiety of **1** (*E*_red_ versus SCE = 0.90 V), which is exergonic in acetone. Electron transfer from OH^−^ to the TAML^•+^ moiety of **1** is expected to be much faster than that to the Co^IV^ moiety of **2**, because the ligand‐centered electron transfer is known to be much faster than the metal‐centered electron transfer.^[^ ^53^, ^54^, ^55^ ^]^ The much faster electron transfer of **1** than that of **2** was confirmed by comparing the electron transfer reactivity of **1** and **2** (vide infra). In the case of **1**, electron transfer from OH^−^ to TAML^•+^ may be faster than the back protonation reaction of OH^−^ to H_2_O, followed by the facile O─O bond formation step (Scheme 2; step iii) to produce H_2_O_2_^•−^. Finally, electron transfer from H_2_O_2_^•−^ to Co^III^ occurs to produce H_2_O_2_ and [(TAML)Co^II^]^2−^ (Scheme 2; step iv), which may be rapidly oxidized by **1** to produce [(TAML)Co^III^]^−^ in accordance with the stoichiometry (Equation (5)). Because the deprotonation of H_2_O is also largely endergonic in acetone, the deprotonation step i) may also be coupled with the electron‐transfer step ii) and the O─O bond formation step iii). This is corroborated by the fact that the presence of excess H^+^ in bulk (from HOTf) does not block the intramolecular deprotonation step at the active site, as also evident from the observed H/D KIE and the huge difference in observed rates between H_2_O and H_2_O_2_.

**Scheme 3:**
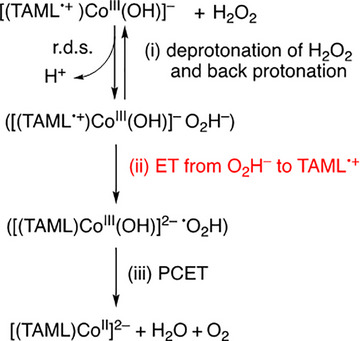
Proposed mechanism for H_2_O_2_ oxidation by [(TAML^•+^)Co^III^(OH)]^−^ **(1)**.

In the case of **2**, however, electron transfer from OH^−^ to the Co^IV^ moiety of **2** may be much slower than the back protonation reaction of OH^−^ to H_2_O. This may be the reason why **1** reacts with H_2_O, but **2** does not react with H_2_O. Similarly, the mechanism of the oxidation of H_2_O_2_ by **1** is proposed as shown in Scheme 3. As in the case of the oxidation of H_2_O by **1** (Scheme 2), the deprotonation of H_2_O_2_ (Scheme 3; step i) may be the rate‐determining step, which is followed by (Scheme 3; step ii) fast exergonic electron transfer from HO_2_^−^ (*E*_ox_ versus SCE = −0.74 V)^[^ ^74^ ^]^ to **1** (*E*_red_ versus SCE = 0.90 V). Then PCET from HO_2_^•^ to [(TAML)Co^III^(OH)]^2−^ (Scheme 3; step iii) occurs to produce O_2_, H_2_O, and [(TAML)Co^II^]^2−^ which is rapidly oxidized by **1** to produce [(TAML)Co^III^]^−^ in accordance with the stoichiometry (Equation (4)). While a minor contribution from HO_2_^•^ disproportionation cannot be completely excluded, we believe that HO_2_^•^ disproportionation process is not predominant. Notably, no second‐order dependence that would be expected for a pathway governed by bimolecular HO_2_^•^ disproportionation is observed. Similarly, no induction period is observed for O_2_ generation; this would be warranted for a mechanism involving accumulation of a freely diffusing radical.

### Electron‐Transfer Reactions of **1** and **2**

**Figure 7:**
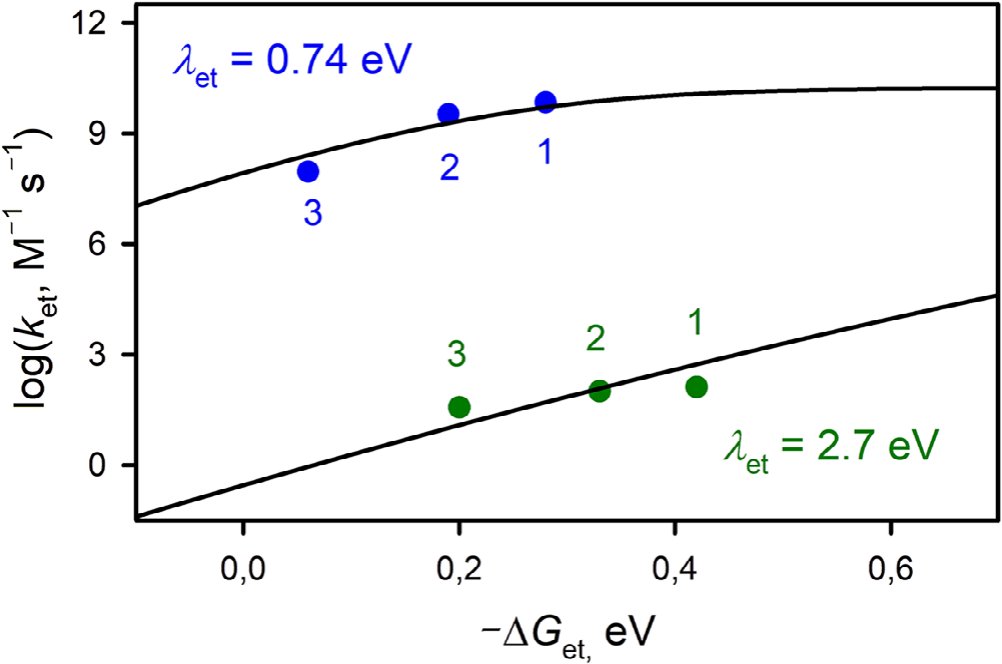
Driving force (–Δ*G*_et_) dependence of log *k*_et_ of ET from electron donors [AcFc (1), Br_2_Fc (2), and Ac_2_Fc (3)] to **1** (blue circles) at 25 °C and to **2** (green circles) at −80 °C in Ar‐saturated acetone.

Both [(TAML^•+^)Co^III^(OH)]^−^ (**1**) and [(H‐TAML)Co^IV^(O)(HOTf)]^−^ (**2**) undergo ET reactions with ferrocene derivatives. First, nanosecond laser‐induced transient absorption measurements were performed to monitor the fast ET from electron donors to **1**, since the ET rate was too fast to be determined with a stopped‐flow technique even at −40 °C. Laser excitation of an Ar‐saturated acetone solution of 9‐mesityl‐10‐methylacridinium ion (Acr^+^‐Mes) at 355 nm resulted in the formation of triplet ET state [^3^(Acr^•^‐Mes^•+^)] (Figure S15a).^[^ ^75^, ^76^, ^77^ ^]^ In the presence of [(TAML)Co^III^]^−^, the decay rate of the absorption band at 490 nm due to the Mes^•+^ moiety of ^3^(Acr^•^‐Mes^•+^) increased with increasing concentration of Li[(TAML)Co]•3H_2_O (Figure S15b). Upon laser photoexcitation at 355 nm, ET from Li[(TAML)Co]•3H_2_O (*E*_ox_ versus SCE = 1.0 V)^[^ ^57^, ^59^, ^60^, ^61^ ^]^ to ^3^(Acr^•^‐Mes^•+^) (*E*_red_ versus SCE = 2.06 V)^[^ ^76^ ^]^ occurred, and a transient absorption band at *λ*_max_ = 595 nm due to **1** was observed (Figure S16a). The transient absorption band at 595 nm due to **1** decayed in the presence of electron donors, such as AcFc, dibromoferrocene (Br_2_Fc), and diacetylferrocene (Ac_2_Fc), indicating the ET from these ferrocene derivatives to **1** (Figure S16b). The second‐order rate constant (*k*_et_) for ET was determined by plotting the pseudo‐first‐order rate constants against the concentrations of electron donors (Figure S17 and Table S11). Additionally, driving force (−∆*G*_et_) dependence of the logarithm of the rate constants (log *k*_et_) of ET from ferrocene derivatives to **1** at 25 °C was also evaluated as shown in Figure 7 (blue circles; see also Figure S18a).

Complex **2** also underwent ET in the presence of electron donors in Ar‐saturated acetone at −80 °C. The *k*_et_ values were determined from the second‐order plot of 1/[(H‐TAML)Co^IV^(O)(HOTf)]^−^ (1/[**2**]) against time for ET from AcFc to **2** in Ar‐saturated acetone at −80 °C (Figure S19 and Table S11). Similarly, *k*_et_ values of ET from other electron donors, such as Br_2_Fc and Ac_2_Fc, to **2** were determined at −80 °C (Figure S20 and Table S11). The −∆*G*_et_ dependence of log *k*_et_ of ET from ferrocene derivatives to **2** at −80 °C is shown in Figure 7 (green circles; see also Figure S18b). We also determined the *k*_et_ values for **2** with Ac_2_Fc at −60 and −70 °C, as shown in Figure S21. Eyring plot (ln(*k*_et_/*T*) versus 1/*T*) for the electron‐transfer reaction of **2** with Ac_2_Fc is shown in Figure S22, where the intercept and slope provided the activation enthalpy (Δ*H* ^‡^ = 5.1 kcal mol^−1−^) and activation entropy (Δ*S* ^‡^ = −24 cal K^−1^ mol^−1^). Using this analysis, the extrapolated *k*_et_ value for **2** at 25 °C is 6.4 × 10^3^ M^−1^ s^−1^ (Figure S23), which is significantly lower than the *k*_et_ value for **1** at 25 °C (9.2 × 10^7^ M^−1^ s^−1^; Figure S17c). This analysis reveals that **1** undergoes electron transfer approximately 1.4 × 10^4^ times faster than **2** at 25 °C, despite **2** being thermodynamically more favored for the electron transfer.

According to the Marcus theory of outer‐sphere ET, the driving force dependence of *k*_et_ is given by Equations (6) and (7),

(6) $$1/k_{\mathrm{et}}=1/k_{\mathrm{diff}}+1/\left[Z_{\exp}\left(-\Delta {G_{\mathrm{et}}}^{\neq }/k_{B}T\right)\right]$$

(7) $$\Delta {G_{\mathrm{et}}}^{\neq }=\left(\lambda_{\mathrm{et}}/4\right){\left(1+\Delta G_{\mathrm{et}}/\lambda_{\mathrm{et}}\right)}^{2}$$

where *k*_diff_ is the diffusion rate constant, Δ*G* ^‡^ is the activation Gibbs energy, and *λ*_et_ is the reorganization energy of ET.^[^ ^65^, ^66^ ^]^ The *λ*_et_ values of **1** and **2** were determined to be 0.74 and 2.7 eV, respectively, with a relatively small experimental error (Figure 7). It has been previously reported that the *λ*_et_ values of ET of Cpd I model complexes with a porphyrin π‐cation radical ligand are between 1.2 and 1.4 eV.^[^ ^53^ ^]^ Thus, the *λ*_et_ value of **1** (0.74 eV) is smaller than the *λ*_et_ values of the ligand‐centered ET of Cpd I model compounds and much smaller than those of the metal‐centered ET of metal(IV)–oxo complexes (e.g., 2.37–2.74 eV).^[^ ^78^, ^79^, ^80^, ^81^, ^82^, ^83^, ^84^, ^85^, ^86^, ^87^, ^88^, ^89^ ^]^ The small *λ*_et_ values were also reported for the ligand‐centered ET of [(TAML^•+^)Fe^V^(NTs)] (1.0 eV)^[^ ^54^ ^]^ and [(TAML^•+^)Cr^V^(O)] (0.26 eV).^[^ ^55^ ^]^ Thus, the small *λ*_et_ value of **1** (0.74 eV) results from the ligand‐centered ET of **1**, whereas the large *λ*_et_ value of **2** (2.7 eV) indicates the metal‐centered ET of **2**. In contrast, the large reorganization energy of **2** with considerable Co(IV) contribution is typical of metal–oxo complexes with a short M–O double bond distance that undergoes a significant elongation upon ET.

## Conclusion

We have shown recently that a cobalt(III)‐hydroxo complex with a one‐electron oxidized TAML ligand [(TAML^•+^)Co^III^(OH)]^−^ (**1**) exists as a distinctive species that is in equilibrium with a cobalt(IV)–oxo species [(H‐TAML)Co^IV^(O)(HOTf)]^−^ (**2**). In the present study, we have shown that **1** reacts with water to produce a stoichiometric amount of O_2_, whereas **2** does not react with H_2_O. We have also shown that the four‐electron oxidation of water by **1** proceeds via two steps, such as the two‐electron oxidation of H_2_O by **1** to yield H_2_O_2_, followed by a much faster two‐electron oxidation of H_2_O_2_ by **1** to produce O_2_. The much higher ET reactivity of **1**, as compared to **2**, has been demonstrated by comparing the driving force dependence of the ET rate constants of **1** and **2**, which affords a small reorganization energy of the ligand‐centered ET of **1** (i.e., 0.74 eV) in contrast to a large reorganization energy of the metal‐centered ET of **2** (i.e., 2.7 eV). We have also demonstrated that **1** is a reactive species in the catalytic oxidation of H_2_O by cobalt(III)‐TAML complex, [(TAML)Co^III^]^−^, with one‐electron oxidants, such as CAN and TBPA^•+^. To the best of our knowledge, this study reports the first example that shows that one of the redox tautomers of a metal‐oxo species oxidizes H_2_O, but the other tautomer shows no reactivity towards H_2_O. The occurrence of ET to the ligand radical cation of **1** in the oxidation of water is also the first time to be observed. Thus, this study has provided valuable new mechanistic insights into the catalytic water oxidation by a metal(III)‐hydroxo complex with a one‐electron oxidized ligand (**1**), in which a much faster ET reduction of **1** than **2** may play the pivotal role for the reaction with H_2_O. A detailed mechanism of the O─O bond formation step, including the Co(III)‐peroxo intermediate, is under investigation in this laboratory.

## Conflict of Interests

The authors declare no conflict of interest.

## Supporting information



Supporting Information

## Data Availability

The data that support the findings of this study are available on request from the corresponding author. The data are not publicly available due to privacy or ethical restrictions.
