# Supplementary material for: Identifying an Active Intermediate and Monitoring O─O Bond Formation in Water Oxidation by a Cobalt(III)‐TAML Complex
Source: Angew Chem Int Ed Engl. 2025 Oct 7;64(48):e202516165. doi: 10.1002/anie.202516165 (PMC12643343; doi:10.1002/anie.202516165)
Supplement: Supplementary file 1 — Supporting Information [file ANIE-64-e202516165-s001.pdf]

Supporting Information

Identifying an Active Intermediate and Monitoring O–O Bond Formation in Water Oxidation by a Cobalt(III)-TAML Complex

Deesha D. Malik,[†] Yong-Min Lee,[†] Shunichi Fukuzumi,*[,†] Kallol Ray,*[,‡] and Wonwoo Nam*[,†,§]

[†] Department of Chemistry and Nano Science, Ewha Womans University, Seoul 03760, Korea

[‡] Department of Chemistry, Humboldt-Universität zu Berlin, Brook-Taylor-Straße 2, 12489 Berlin, Germany

[§] College of Chemistry and Chemical Engineering, Yan'an University, Yan'an, Shaanxi Province, 716000 P. R. China

*Correspondence E-mails: wwnam@ewha.ac.kr; kallol.ray@cms.hu-berlin.de; fukuzumi@chem.eng.osaka-u.ac.jp

Table of Contents

**Experimental Section**	S4
Materials	S4
Instrumentation	S4
Generation of Cobalt-Oxygen Species	S5
Kinetic Studies of **1** and **2**	S5
Dioxygen Quantification by GC	S6
Dioxygen Labeling Experiment by GC-MS	S7
Detection of H_2O_2 using Ti-TPyP	S7
References	S8
Table S1	S9
Table S2	S10
Table S3	S11
Table S4	S12
Table S5	S13
Table S6	S14
Table S7	S15
Table S8	S16
Table S9	S17
Table S10	S18
Table S11	S19
Table S12	S20
Figure S1	S21
Figure S2	S22
Figure S3	S23
Figure S4	S24
Figure S5	S25
Figure S6	S26
Figure S7	S27
Figure S8	S28
Figure S9	S29
Figure S10	S30
Figure S11	S31
Figure S12	S32

Figure S13	S33
Figure S14	S34
Figure S15	S35
Figure S16	S36
Figure S17	S37
Figure S18	S38
Figure S19	S39
Figure S20	S40
Figure S21	S41
Figure S22	S42
Figure S23	S43
Figure S24	S44

Experimental Section

Materials. All chemicals, such as triflic acid, cerium(IV) ammonium nitrate (CAN), and tris(4-bromophenyl)ammonium hexachloroantimonate radical cation ($TBPA^{\bullet+}$), which were of the best available purity, were purchased from Sigma-Aldrich Chemical Co. Solvents were dried according to the published procedures prior to use.[S1] H_4TAML (H_4TAML = 3,4,8,9-tetrahydro3,3,6,6,9,9-hexamethyl-1H-1,4,8,11-benzotetraazo-cyclotridecane2,5,7,10-(6H,11H)-tetrone) was purchased from GreenOx Catalyst Inc. (Pittsburgh, PA).[S2] Iodosylbenzene (PhIO) was prepared by following the literature method.[S3] The starting cobalt(III) complex, Li[(TAML)Co^{III}]•3H_2O, was synthesized by following the literature procedure with readily available starting materials.[S4,S5] $H_2{}^{18}O$ (98% ^{18}O-enriched) was purchased from Cambridge Isotope Inc. (USA). Water (18.2 MΩ cm) was purified with Milli-Q system (Millipore; Milli-Q Jr).

Instrumentation. UV–vis spectra were recorded on a Hewlett-Packard 8453 diode array spectrophotometer equipped with a UNISOKU Scientific Instruments USP-203A Cryostat for low-temperature experiments or on a UNISOKU RSP-601 stopped-flow spectrometer equipped with a MOS-type highly sensitive photodiode array detector. Nanosecond time-resolved transient absorption measurements were performed using an Nd:YAG laser as follows. A solution mixture in a quartz cell (1.0 cm × 1.0 cm) was excited by a Nd:YAG laser (Continuum SLII-10, 4–6 ns fwhm, λ_{ex} = 355 nm, 80 mJ $pulse^{-1}$, 10 Hz). Cold spray ionization time-of-flight mass spectra (CSI-MS) were collected on a JMS-T100CS (JEOL) mass spectrometer equipped with the CSI source. Typical measurement conditions are as follows: Needle voltage: 2.2 kV, office 1 current: 50 – 500 nA, office1 voltage: 0 to 20 V, ringlens voltage: 10 V, ion source temperature: 0 °C, spray temperature: –40 °C. The CSI-MS spectra of **1** with water was taken by directly infusing the reaction solution into the ion source through a pre-cooled tube under high N_2 gas pressure. The samples were directly infused into the source at 20 μL/min to collect electrospray ionization mass (ESI MS) spectra on a Thermo Finnigan (San Jose, CA, USA.) LCQTM Advantage MAX quadrupole ion trap instrument. The spray voltage was set at 3.7 kV and the capillary temperature at 80 °C. The solution electron paramagnetic resonance (EPR) spectra were measured at 25 °C and –40 °C using a JEOL X-band spectrometer (JES-

FA100).[S6] The experimental parameters for EPR measurements by JES-FA100 were as follows: Microwave frequency = 9.028 GHz, microwave power = 1.0 mW, modulation amplitude = 1.0 mT, modulation frequency = 100 kHz, and time constant = 0.03 s. The *g* value was calibrated using the Mn^{2+} marker. Dioxygen quantification was carried out with Shimadzu GC-2030 gas chromatograph (GC). Mass spectra monitoring O_2 isotopes [32 (^{16}O–^{16}O), 34 (^{16}O–^{18}O), and 36 (^{18}O–^{18}O)] were analyzed by gas chromatography–mass spectrometry (GC-MS; a Shimadzu GC-17A gas chromatograph equipped with a Shimadzu QP-5000 mass spectrometer) at 40°C. Cyclic voltammetry (CV) measurements were performed on a CHI630B electrochemical analyzer (CH Instruments, Inc.) under an Ar atmosphere in deaerated acetone containing nBu$_4$NPF$_6$ (TBAPF$_6$; 0.10 M) as the supporting electrolyte at 0 °C for **1** and −40 °C for **2**. A conventional three-electrode cell was used with a glassy carbon working electrode (surface area of 0.30 mm^2), a platinum wire as a counter electrode and an Ag/AgNO$_3$ (0.010 M) electrode as a reference electrode. All potentials [vs. Ag/AgNO$_3$ (0.010 M)] were converted to values versus SCE by adding 0.29 V.

Generation of Cobalt-Oxygen Species. Intermediate [(TAML$^{\bullet+}$)Co^{III}(OH)]$^{-}$ (**1**) was prepared by reacting Li[(TAML)Co^{III}]•3H$_2$O with PhIO (0.50 equiv.), followed by the addition of HOTf (0.50 equiv.) in Ar-saturated acetone at 25 ^{o}C. **1** was also prepared by reacting Li[(TAML)Co^{III}]•3H$_2$O with 1.0 equiv. of CAN or TBPA$^{\bullet+}$ in Ar-saturated acetone at 25 ^{o}C. For CSI-MS measurement, ^{18}O-labeled [(TAML)Co(^{18}O)(H)]$^{-}$ (**1**-^{18}O) was synthesized by incubating PhIO (1.0 equiv.) in the presence of H$_2$^{18}O (5.0 μL) to generate PhI^{18}O prior to its reaction with Li[(TAML)Co^{III}]•3H$_2$O and HOTf (0.50 equiv.) in Ar-saturated acetone. For solution EPR studies the intermediates, [(TAML$^{\bullet+}$)Co^{III}(OH)]$^{-}$ (**1**) and [(H-TAML)Co^{IV}=O(HOTf)]$^{-}$ (**2**), were prepared by reacting Li[(TAML)Co^{III}]•3H$_2$O with PhIO (3.0 equiv.), followed by the addition of HOTf (5.0 equiv.) in Ar-saturated acetone at –40 ^{o}C. For CV, **1** and **2** were generated upon addition of PhIO (3.0 equiv.) and HOTf (5.0 equiv.) to Ar-saturated acetone solution of Li[Co^{III}(TAML)]·3H$_2$O (2.0 mM) at 0 ^{o}C and –40 ^{o}C, respectively.

Kinetic Studies of 1 and 2. All reactions were run at least in triplicate, and the data reported represents the average of these reactions. Kinetic measurements were performed on a Hewlett

Packard 8453 photodiode-array spectrophotometer at 25 ^{o}C. For the reactions of **1** with H_2O or D_2O, k_2 was determined under pseudo-first-order conditions by fitting the changes in absorbance at 600 nm due to **1**. For the ET reactions by **2** with ferrocene derivatives (AcFc, Br_2Fc, and Ac_2Fc), k_{et} was determined under pseudo-first-order conditions by fitting the changes in absorbance at 950 nm due to **2** in acetone at –80 ^{o}C. Nanosecond laser-induced transient absorption measurements were performed to examine the fast ET from the electron donors (AcFc, Br_2Fc, and Ac_2Fc) to **1** in acetone at 25 ^{o}C. **1** was generated in situ by ET from Li[Co(TAML)]•3H_2O to the triplet ET state of 9-mesityl-10-methylacridinium ion (^{3}Acr$^•$-Mes$^{•+}$), where the transient absorption band due to Mes$^{•+}$ moiety of ^{3}(Acr$^•$-Mes$^{•+}$) (λ_{max} = 490 nm) and the absorption band due to **1** (λ_{max} = 595 nm) were observed upon laser photoexcitation. Then ET from the electron donors to **1** was monitored by the decay of absorbance at 595 nm due to **1**.

Dioxygen Quantification by GC. Dioxygen quantification was performed with a Shimadzu GC-2030 gas chromatograph (GC). The yield of dioxygen in the water oxidation reaction by **1** was carried out in presence of various oxidants such as CAN, TBPA$^{•+}$, and PhIO/HOTf under stoichiometric and catalytic conditions. The stoichiometric water oxidation reaction by **1** was monitored in the presence of Li[(TAML)Co^{III}]•3H_2O and PhIO/HOTf (or CAN) in Ar-saturated acetone at 25 ^{o}C. A 5.0 mL long vial containing Li[(TAML)Co^{III}]•3H_2O in 2.0 mL acetone was purged under argon for 30 min. PhIO, HOTf, CAN and water were purged separately under argon for 15 min. Purged PhIO (0.50 equiv.) and HOTf (0.50 equiv.) were simultaneously added to the reaction vial via syringe to generate **1**, followed by addition of 0.50 M water. Similarly, purged CAN (1.0 equiv.) was added to the reaction vial containing Li[(TAML)Co^{III}]•3H_2O via syringe to generate **1**, followed by addition of 0.50 M water. After 10 mins, 100 µL of gas was injected from the headspace of the reaction vial into the GC. Catalytic water oxidation by **1** in acetone was monitored in the presence of CAN and TBPA$^{•+}$. Reaction solution containing CAN (or TBPA$^{•+}$) and water was purged under argon for 15 min. The oxidant solution was injected into the purged vial containing Li[(TAML)Co^{III}]•3H_2O, followed by addition of water (0.50 M) via a gas-tight syringe. After 5 min, 100 µL of gas in the headspace of the sample vial was

injected into the GC. Products were quantified by making the calibration plot using a known concentration of saturated dioxygen in acetone (Figure S23 and Table S12).

Dioxygen Labeling Experiment by GC-MS. ^{18}O-labeled dioxygen was analyzed by using GC-MS instrument with Pfeffer vacuum (model GSD 320) with O_2 mass spectrometer. A 5.0 mL long vial containing Li[(TAML)Co^{III}]•3H_2O (0.05 mM) and $H_2{}^{18}O$ (1.0 M) was sealed with a rubber septum and purged under nitrogen for 30 min and then connected to the GC-MS and kept under vacuum for 8 – 10 h. After the vacuum stabilization, a nitrogen purged solution of $TBPA^{•+}$ (5.0 mM) was added to the reaction vial containing Li[(TAML)Co^{III}]•3H_2O and $H_2{}^{18}O$ via a gas-tight syringe. Following the in-situ generation of **1**-^{18}O and its subsequent reaction with $H_2{}^{18}O$, a notable increase in the peak intensity at m/z = 36, corresponding to $^{18}O_2$, was observed immediately, indicating the rapid formation of $^{18}O_2$ during the reaction.

Detection of H_2O_2 using Ti-TPyP. An aqueous solution of the oxo-[5,10,15,20-tetra(4-pyridyl)porphyrinato]titanium(IV) (Ti-TPyP) complex is an exceptionally sensitive reagent for detecting hydrogen peroxide (H_2O_2).[S7] The interaction of H_2O_2 with the Ti-TPyP complex results in noticeable spectral changes, which were monitored using UV-vis spectroscopy. The Ti-TPyP complex exhibits a Soret band at 434 nm (Figure S24 and Table S12), which diminishes in intensity upon the addition of H_2O_2.[S7] This decrease in absorbance at 434 nm is directly proportional to the concentration of H_2O_2 present. The concentration of H_2O_2 produced in the reaction was quantified through spectroscopic titration with the Ti-TPyP reagent. To prepare the reagent, 50 μM of Ti-TPyP was dissolved in 50 mM hydrochloric acid in water (HCl). For each sample, a mixture containing 250 μL of the Ti-TPyP reagent (50 μM) and 250 μL of perchloric acid in water (4.8 M) was added to 250 μL of the sample solution. This solution was left to stand at room temperature for 5 min. Following this incubation, the solution was diluted to a final volume of 2.5 mL by adding 1.75 mL of water in a UV-vis cuvette with a 1.0 cm path length, and the absorbance at 434 nm was measured. A blank solution was prepared in a similar manner, using the corresponding solvent (250 μL) instead of the sample solution. A decrease in absorbance at 434 nm, when compared to the blank solution, indicates the presence of H_2O_2 in the sample. The H_2O_2 concentration in the sample solution was determined by

comparing the observed absorbance to those obtained from known concentrations of authentic H_2O_2 solutions.

References

[S1] W. L. F. Armarego, C. L. L. Chai, Purification of Laboratory Chemicals, 6th ed., Pergamon Press, Oxford, **2009**.

[S2] C. P. Horwitz, A. Ghosh, US Patent 7060818, **2006**.

[S3] H. Saltzman, J. G. Sharefkin, Org. Synth. **1973**, *43*, 658.

[S4] D. D. Malik, W. Ryu, Y. Kim, G. Singh, J.-H. Kim, M. Sankaralingam, Y.-M. Lee, M. S. Seo, M. Sundararajan, D. Ocampo, M. Roemelt, K. Park, S. H. Kim, M.-H. Baik, J. Shearer, K. Ray, S. Fukuzumi, W. Nam, *J. Am. Chem. Soc.* **2024**, *146*, 13817–13835.

[S5] T. J. Collins, R. D. Powell, C. Slebodnick, E. S. Uffelman, *J. Am. Chem. Soc.* **1991**, *113*, 8419–8425.

[S6] M. Ju, J. Kim, J. Shin, *Bull. Korean Chem. Soc.* **2024**, *45*, 835-862.

[S7] C. Matsubara, N. Kawamoto, K. Takamura, *Analyst* **1992**, *117*, 1781–1784.

Table S1. Catalytic water oxidation by $[(TAML)Co^{III}]^-$ with various concentrations of $TBPA^{\bullet+}$ in Ar-saturated acetone at 25 °C.[a]

$[(TAML)Co^{III}]^-$ (μM)	H_2O (M)	$TBPA^{\bullet+}$ (mM)	O_2 (mM)	O_2 yield (%)	TON	Total TON
0.0	0.50	1.0	-	-	-	-
200	0.50	1.0	0.27	27	1.3	5.2
200	0.50	2.0	0.40	21	2.0	8.0
200	0.50	3.0	0.60	19	3.0	12
100	0.50	1.0	0.20	19	2.0	8.0
100	0.50	2.0	0.40	20	4.0	16
100	0.50	3.0	0.60	21	6.0	24
100	0.50	5.0	1.1	21	11	44
100	0.50	10	2.0	20	20	80
50	0.50	1.0	0.20	21	4.0	16
50	0.50	2.0	0.40	20	8.0	32
50	0.50	4.0	1.1	29	23	92
50	0.50	6.0	1.5	25	30	120

[a] The catalytic water oxidation reaction was performed with different concentrations of $Li[Co^{III}(TAML)]\cdot 3H_2O$ (200, 100, and 50 μM) and $TBPA^{\bullet+}$ (1.0 – 6.0 mM) in Ar-saturated acetone at 25 °C. Yield of O_2 and turnover number (TON) were calculated based on the concentration of $TBPA^{\bullet+}$ and the catalyst concentration, respectively. Total TON was obtained by multiplying the TON by 4, corresponding to the four-electron oxidation of water to produce O_2 (see Equations (1) and (2) in text).

Table S2. Catalytic water oxidation by $[(TAML)Co^{III}]^-$ with various concentrations of CAN in Ar-saturated acetone at 25 °C.[a]

$[(TAML)Co^{III}]^-$ (μM)	H_2O (M)	CAN (mM)	O_2 (mM)	O_2 yield (%)	TON	Total TON
0.0	0.50	0.75	-	-	-	-
100	0.50	0.75	0.20	26	2.0	8.0
100	0.50	1.0	0.26	26	3.0	12
100	0.50	1.5	0.40	25	4.0	16
100	0.50	3.0	0.65	22	7.0	28
100	0.50	5.0	1.0	19	10	40
100	0.50	10	1.8	18	18	72

[a] Yield of O_2 was determined based on the concentration of CAN. TON was calculated based on the catalyst concentration ($[O_2]/[[(TAML)Co^{III}]^-]$). Total TON was obtained by multiplying the TON by 4, corresponding to the four-electron oxidation of water to produce O_2 (see Equations (1) and (2) in text).

Table S3. Catalytic water oxidation by $[(TAML)Co^{III}]^-$ and CAN with different concentrations of $[(TAML)Co^{III}]^-$ in Ar-saturated acetone at 25 °C.[a]

$[(TAML)Co^{III}]^-$ (μM)	H_2O (M)	CAN (mM)	O_2 (mM)	O_2 yield (%)	TON	Total TON
0.0	0.50	10	-	-	-	-
50	0.50	10	1.2	12	23	92
30	0.50	10	0.75	7.5	25	100
20	0.50	10	0.53	5.3	26	104
10	0.50	10	0.40	4.0	40	160
5.0	0.50	10	0.33	3.3	66	264

[a] Yield of O_2 was calculated based on the concentration of CAN. TON was calculated based on the catalyst concentration. Total TON was obtained by multiplying the TON by 4, corresponding to the four-electron oxidation of water to produce O_2 (see Equations (1) and (2) in text).

Table S4. Catalytic water oxidation by $[(TAML)Co^{III}]^{-}$ and $TBPA^{•+}$ with different concentrations of $[(TAML)Co^{III}]^{-}$ in Ar-saturated acetone at 25 °C.[a]

$[(TAML)Co^{III}]^{-}$ (μM)	H_2O (M)	$TBPA^{•+}$ (mM)	O_2 (mM)	O_2 yield (%)	TON	Total TON
0.0	0.50	5.0	-	-	-	-
100	0.50	5.0	1.4	27	14	56
50	0.50	5.0	1.3	26	26	104
25	0.50	5.0	1.2	24	48	192
20	0.50	5.0	1.1	22	55	220
10	0.50	5.0	1.0	25	100	400
5.0	0.50	5.0	0.75	15	150	600

[a] Yield of O_2 was calculated based on the initial concentration of $TBPA^{•+}$. TON was calculated based on the catalyst concentration. Total TON was obtained by multiplying the TON by 4, corresponding to the four-electron oxidation of water to produce O_2 (see Equations (1) and (2) in text).

Table S5. Initial rates of the catalytic oxidation of H_2O by $[(TAML)Co^{III}]^-$ and $TBPA^{\bullet+}$ in Ar-saturated acetone at 25 °C.[a]

$[(TAML)Co^{III}]^-$ (μM)	$-d[TBPA^{\bullet+}]/dt$
10	5.0×10^{-4}
20	1.5×10^{-3}
50	4.5×10^{-3}
100	8.5×10^{-3}

[a] The catalytic water oxidation reaction was performed with different concentrations of $Li[Co^{III}(TAML)]\cdot 3H_2O$ (10 – 100 μM) in the presence of $TBPA^{\bullet+}$ (0.50 mM) and water (0.50 M) in Ar-saturated acetone at 25 °C.

Table S6. Water oxidation by **1** prepared with CAN in Ar-saturated acetone at 25 °C.[a]

$[(TAML)Co^{III}]^-$ (mM)	H_2O (M)	CAN (mM)	O_2 (mM)	O_2 yield (%)
0.50	0.50	0.50	0.11	22
0.75	0.50	0.75	0.19	25
1.0	0.50	1.0	0.25	25
1.5	0.50	1.5	0.34	23
2.0	0.50	2.0	0.49	25

[a] **1** was generated by reacting $Li[Co^{III}(TAML)]\cdot 3H_2O$ (0.50 – 2.0 mM) with CAN (1.0 equiv.) and then reacted with water (0.50 M) in Ar-saturated acetone at 25 °C. Yield of O_2 was calculated based on the $Li[Co^{III}(TAML)]\cdot 3H_2O$ concentration (see Equation (3) in text).

Table S7. Water oxidation by **1** prepared with PhIO and HOTf in Ar-saturated acetone at 25 °C.[a]

$[(TAML)Co^{III}]^-$ (mM)	H_2O (M)	PhIO (mM)	HOTf (mM)	O_2 (mM)	O_2 yield (%)
0.50	0.50	0.25	0.25	0.12	24
1.0	0.50	0.50	0.50	0.24	24
1.5	0.50	0.75	0.75	0.38	25
2.0	0.50	1.0	1.0	0.48	24

[a] **1** was generated by reacting $Li[Co^{III}(TAML)]\cdot 3H_2O$ (0.50 – 2.0 mM) with PhIO (0.50 equiv.) and HOTf (0.50 equiv.) and then reacted with water (0.50 M) in Ar-saturated acetone at 25 °C. Yield of O_2 was calculated based on the $Li[Co^{III}(TAML)]\cdot 3H_2O$ concentration (see Equation (3) in text).

Table S8. Oxidation of H_2O_2 by **1** to produce O_2 in Ar-saturated acetone at 25 °C.[a]

**1** (mM)	H_2O_2 (mM)	O_2 (mM)
0.50	0.25	0.18
1.0	0.50	0.45
1.5	0.75	0.60
2.0	1.0	0.80

[a] **1** was generated by reacting Li[Co^{III}(TAML)]·$3H_2O$ (0.50 – 2.0 mM) with CAN (1.0 equiv.) and then reacted with H_2O_2 (0.50 equiv.) in Ar-saturated acetone at 25 °C (see Equation (4) in text).

Table S9. Oxidation of water (15 M) by **1** to produce H_2O_2 in Ar-saturated acetone at 25 ^oC.[a]

**1** (μM)	H_2O_2 (μM)	total yield of H_2O_2 (%)
10	4.4	88
20	8.4	84
50	20	80
100	39	78
150	48	64

[a] **1** was generated by reacting Li[Co^{III}(TAML)]·3H_2O (10 – 150 μM) with CAN (1.0 equiv.) and then reacted with water (15 M) in Ar-saturated acetone at 25 ^oC. Total yield (%) of H_2O_2 was calculated based on the two-electron oxidation of water (see Equation (5) in text).

Table S10. Oxidation of water by **1** to produce H_2O_2 in the presence of various concentrations of water in Ar-saturated acetone at 25 ^oC.[a]

**1** (μM)	H_2O (M)	H_2O_2 (μM)	total yield of H_2O_2 (%)
20	1.0	0.0	0
20	3.0	0.0	0
20	5.0	2.4	24
20	7.0	2.7	27
20	10	5.1	51
20	15	8.4	84

[a] **1** was generated by reacting Li[Co^{III}(TAML)]·$3H_2O$ (20 μM) with CAN (1.0 equiv.) and then reacted with various concentrations of water in Ar-saturated acetone at 25 ^oC. Total yield (%) of H_2O_2 was calculated based on the two-electron oxidation of water (see Equation (5) in text).

Table S11. One-electron oxidation potentials (E_{ox}) of electron donors, rate constants (k_{et}), and driving force ($-\Delta G_{et}$) of ET from electron donors to **1** at 25 °C and **2** at –80 °C in Ar-saturated acetone.[a]

electron donor	E_{ox} vs. SCE, V	**1**		**2**	
		$-\Delta G_{et}$, eV	k_{et}, M^{-1} s^{-1}	$-\Delta G_{et}$, eV	k_{et}, M^{-1} s^{-1}
AcFc	0.62	0.28	$7.0(5) \times 10^9$	0.42	$1.3(1) \times 10^2$
Br_2Fc	0.71	0.19	$3.3(2) \times 10^9$	0.33	$1.1(1) \times 10^2$
Ac_2Fc	0.84	0.06	$9.2(6) \times 10^7$	0.21	$3.7(3) \times 10^1$

[a] Fast ET from electron donors (AcFc, Br_2FC and Ac_2Fc) to **1** was investigated using nanosecond laser-induced transient absorption measurements at 25 °C. **1** was generated in situ by ET from Li[Co(TAML)]·$3H_2O$ (0.010 mM) to the triplet ET state of 9-mesityl-10-methylacridinium ion ($^3Acr^{\bullet}$-$Mes^{\bullet+}$, 0.20 mM) in Ar-saturated acetone at 25 °C. Intermediate **2** was generated by reacting Li[Co^{III}(TAML)]·$3H_2O$ (1.0 mM) with PhIO (3.0 equiv.) and HOTf (5.0 equiv.) and then reacted with various electron donors (AcFc, Br_2FC and Ac_2Fc) in Ar-saturated acetone at –80 °C. (see above ‘Kinetic Studies of **1** and **2**’ section in Experimental Section for details).

Table S12. O_2 and H_2O_2 calibration data using authentic H_2O_2 and O_2 in Ar-saturated acetone.[a]

O_2 (mM)	area of O_2	H_2O_2 (μM)	ΔA_{434} nm
0.30	2.9×10^3	5.0	0.15
0.60	4.8×10^3	10	0.30
0.90	7.3×10^3	15	0.53
1.2	1.0×10^4	20	0.74
1.5	1.3×10^4		
1.8	1.6×10^4		
2.1	1.9×10^4		

[a] O_2 calibration was performed using GC by adding known concentrations of O_2-saturated acetone to Ar-saturated acetone at 25 °C. H_2O_2 calibration was conducted using the Ti-TPyP reagent, with measurements taken by UV-vis spectroscopy at 434 nm in Ar-saturated acetone at 25 °C (see Experimental Section for details).

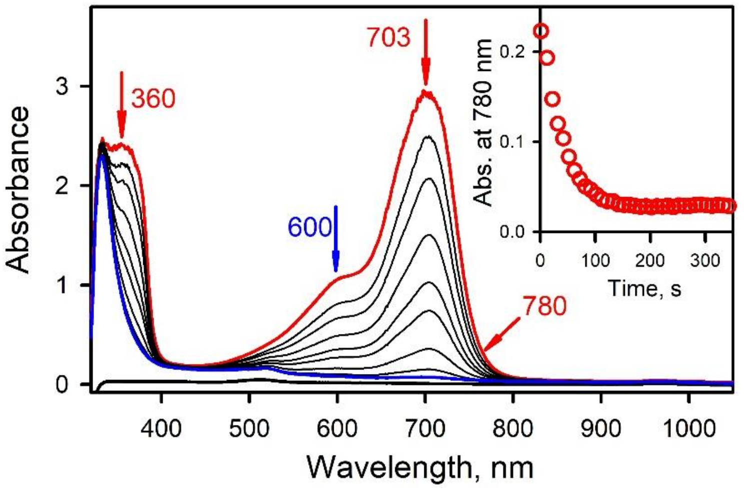

Figure S1. UV-vis spectral change recorded during the catalytic water oxidation by **1** in Ar-saturated acetone containing Li[Co^{III}(TAML)]·3H_2O (0.010 mM), $TBPA^{•+}$ (0.50 mM), and water (0.50 M) at 25 °C. Inset shows time course monitored at 780 nm.

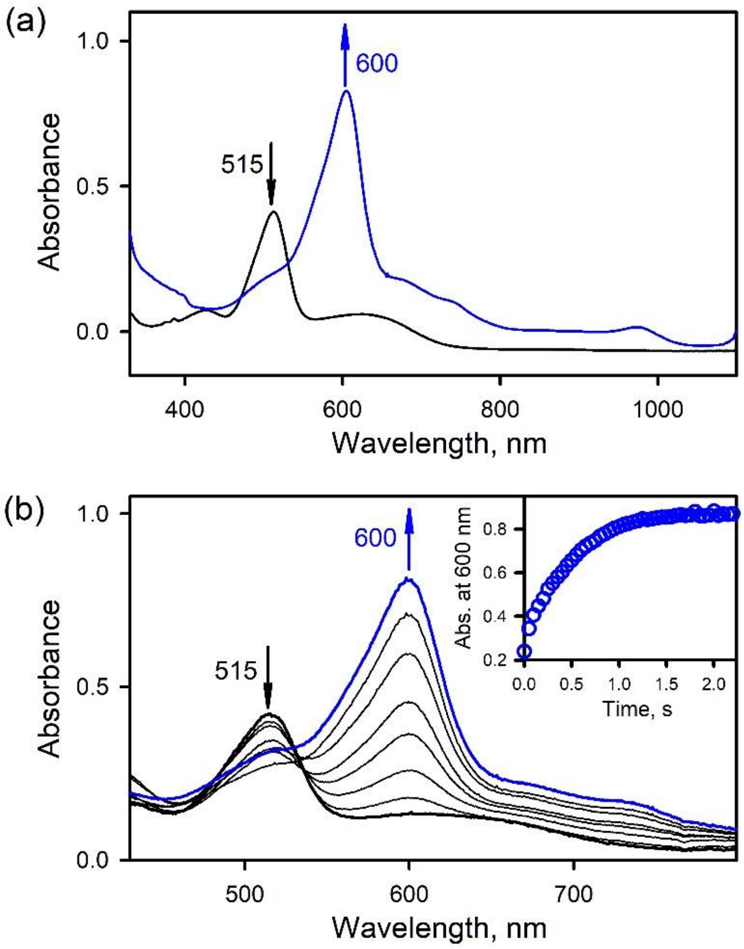

Figure S2. (a) UV-vis spectra showing the generation of intermediate **1** (blue line) upon addition of PhIO (0.50 equiv.) and HOTf (0.50 equiv.) to Li[Co^{III}(TAML)]·3H_2O (0.20 mM; black line) in Ar-saturated acetone at 25 ^oC. (b) Stopped flow UV-vis spectral changes showing the generation of **1** (blue line) upon addition of CAN (1.0 equiv.) into Ar-saturated acetone solution of Li[Co^{III}(TAML)]·3H_2O (0.10 mM; black line) at 25 ^oC. Inset shows time trace monitored at 600 nm due to **1**.

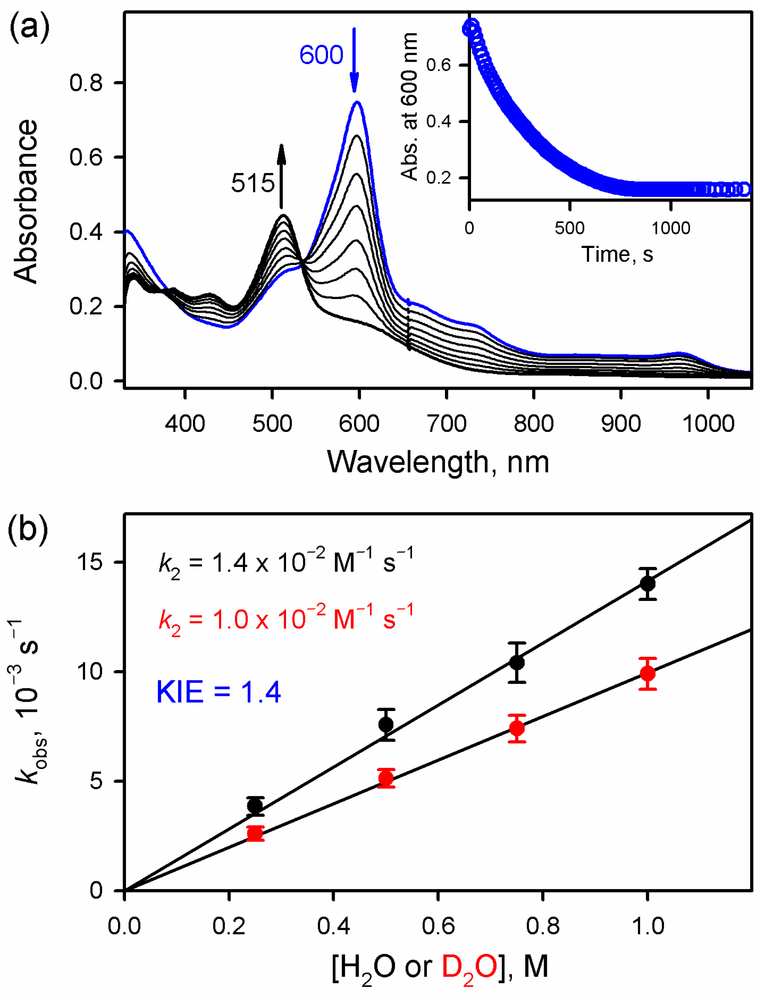

Figure S3. (a) UV−vis spectral changes showing the decay of **1** (blue line) to $[(TAML)Co^{III}]^{-}$ (black line) upon addition of water (1.0 M) to **1** (0.10 mM) in Ar-saturated acetone at 25 °C. Inset shows time trace monitored at 600 nm due to **1**. (b) Plots of pseudo-first-order rate constants vs. concentrations of H_2O (black circles) and D_2O (red circles) in water oxidation by **1** in Ar-saturated acetone at 25 °C. **1** was prepared by ET oxidation of $Li[Co^{III}(TAML)]\cdot 3H_2O$ (0.10 mM) with CAN (1.0 equiv.) in Ar-saturated acetone at 25 °C.

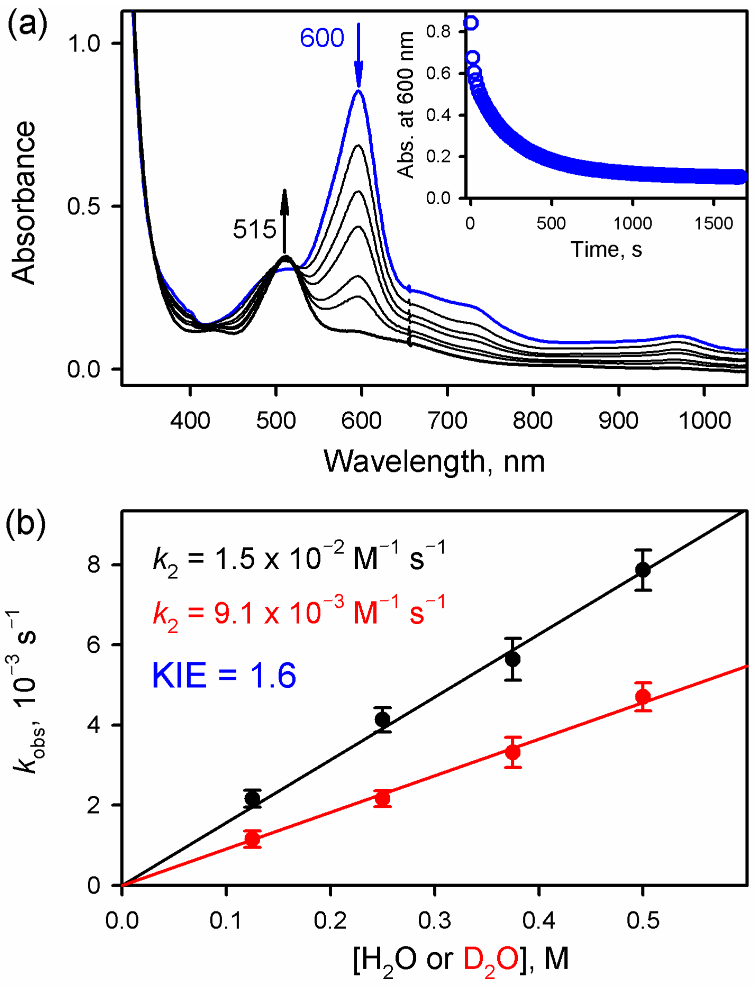

Figure S4. (a) UV−vis spectral changes showing the decay of **1** (blue line) to $[(TAML)Co^{III}]^-$ (black line) upon addition of water (1.0 M) to **1** (0.10 mM) in Ar-saturated acetone at 25 °C. Inset shows time course monitored at 600 nm due to **1**. (b) Plots of pseudo-first-order rate constants vs. concentrations of H_2O (black circles) and D_2O (red circles) in water oxidation by **1**. Intermediate **1** was produced by ET oxidation of $Li[Co^{III}(TAML)]\cdot 3H_2O$ (0.10 mM) with $TBPA^{\bullet+}$ (1.0 equiv.) in Ar-saturated acetone at 25 °C.

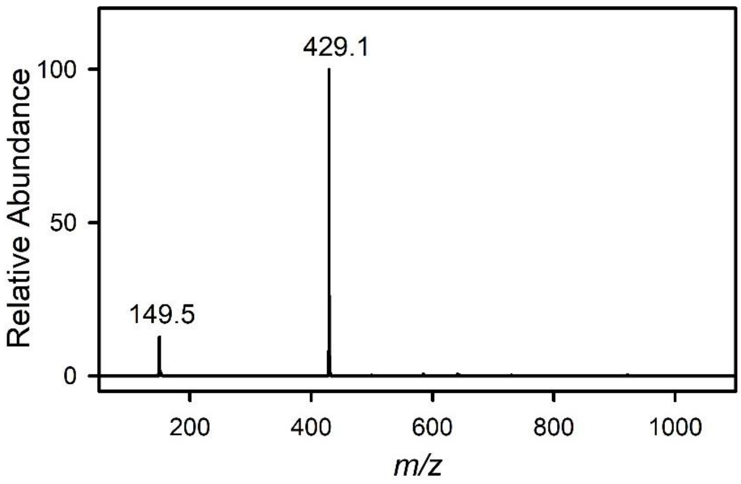

Figure S5. ESI-MS spectrum of the decayed species in the water oxidation reaction catalyzed by **1** (0.10 mM) in the presence of water (0.50 M) in Ar-saturated acetone at 25 °C. The peaks at *m/z* = 429.1 and 149.5 correspond to $[(TAML)Co^{III}]^{-}$ (calcd. *m/z* = 429.1) and OTf^{-} (calcd. *m/z* = 149.5), respectively. **1** was generated by reacting $Li[Co^{III}(TAML)]\cdot 3H_2O$ (0.10 mM) with PhIO (0.50 equiv.) and HOTf (0.50 equiv.) and then reacted with water (0.50 M) in Ar-saturated acetone at 25 °C.

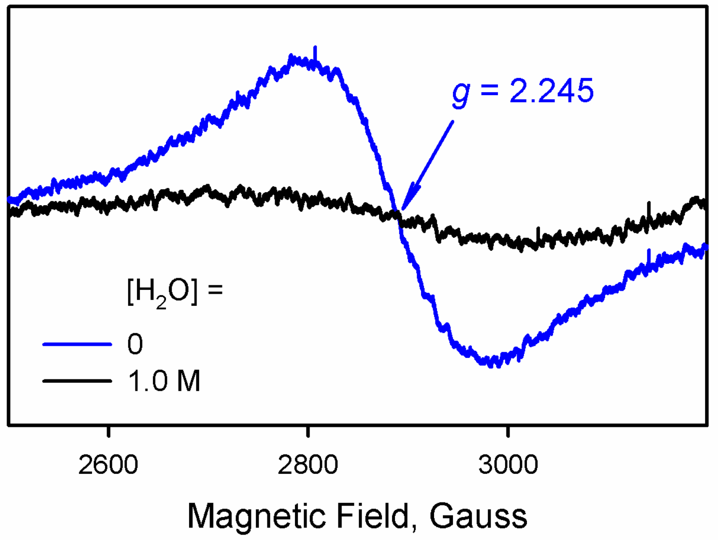

Figure S6. Solution EPR spectra of **1** (4.0 mM) in the absence (blue line) and presence of water (1.0 M; black line) in Ar-saturated acetone at 0 °C. **1** was generated by reacting Li[Co^{III}(TAML)]·3H_2O (4.0 mM) with PhIO (0.50 equiv.) and HOTf (0.50 equiv.) and then reacted with water (1.0 M; black line) in Ar-saturated acetone at 25 °C.

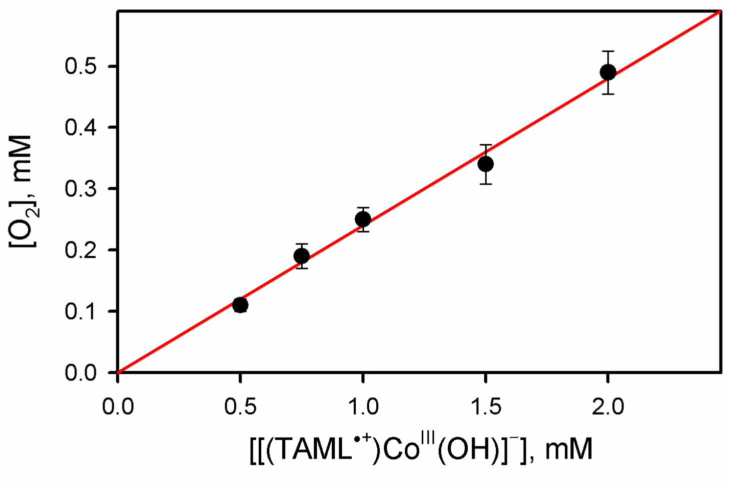

Figure S7. Plot of concentration of **1** vs. concentration of O_2 produced in the oxidation of water (0.50 M) by **1** (0.50 – 2.0 mM) in Ar-saturated acetone at 25 °C. **1** was generated by the ET oxidation of Li[Co(TAML)]•3H_2O (0.50 – 2.0 mM) with CAN (1.0 equiv.) in Ar-saturated acetone at 25 °C (see Table S6).

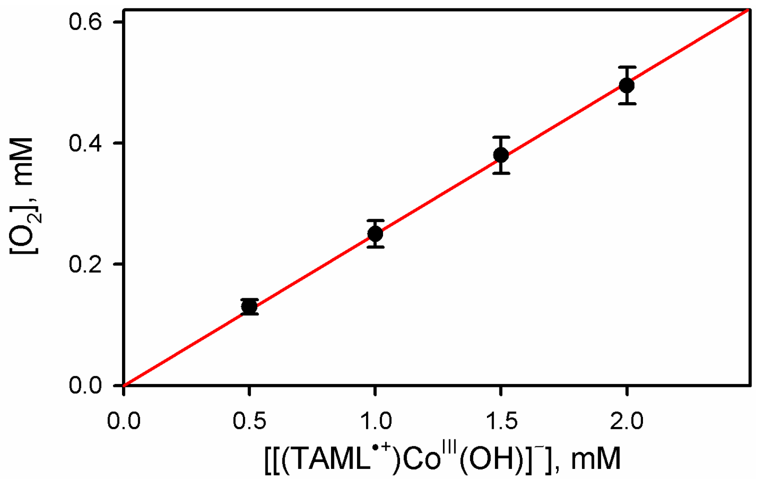

Figure S8. Plot of concentration of **1** vs. concentration of O_2 produced in the oxidation of water (0.50 M) by **1** (0.50 – 2.0 mM) in Ar-saturated acetone at 25 °C. **1** was generated by the oxidation of Li[Co(TAML)]·3H_2O (0.50 – 2.0 mM) with PhIO (0.50 equiv.) and HOTf (0.50 equiv.) in Ar-saturated acetone at 25 °C.

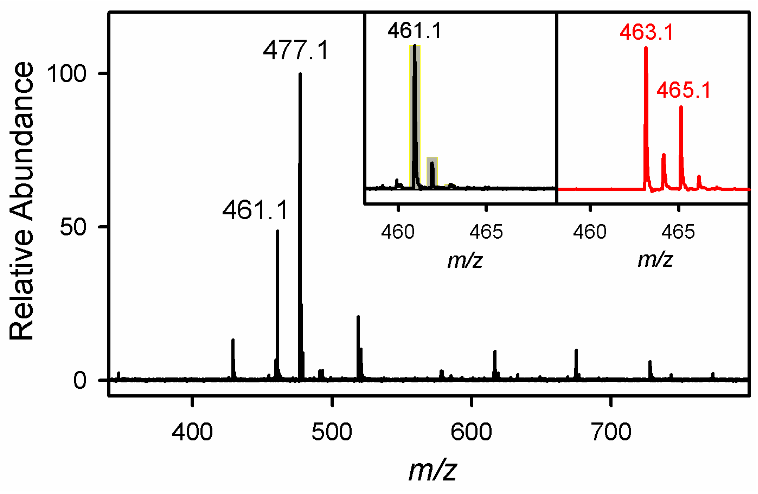

Figure S9. Negative mode CSI-MS spectrum taken in the water oxidation reaction by **1** upon addition of $H_2{}^{16}O$ to the solution of **1**. **1** was produced by reacting Li[(TAML)Co^{III}]·$3H_2O$ (0.10 mM) with PhIO (1.0 equiv.) and HOTf (0.50 equiv.) in Ar-saturated acetone at –40 °C. The peaks at *m/z* = 461.1 and 477.1 correspond to $[(TAML)Co(OO)]^-$ (calcd. *m/z* = 461.1) and $[(CH_3CN)(Li)(TAML)Co]^-$ (calcd. *m/z* = 477.1), respectively. Insets show the observed isotope distribution patterns for $[(TAML)Co(^{16}O^{16}O)]^-$ (*m/z* = 461.1) originated from **1**-^{16}O with $H_2{}^{16}O$ (left panel, 20 µL, black line) and $[(TAML)Co(^{16}O^{18}O)]^-$ (*m/z* = 463.1) from **1**-^{16}O with $H_2{}^{18}O$ and $[(TAML)Co(^{18}O^{18}O)]^-$ (*m/z* = 465.1) from **1**-^{18}O with $H_2{}^{18}O$ (right panel, 20 µL, red line). Yellow bars in left inset show the calculated isotope distribution patterns.

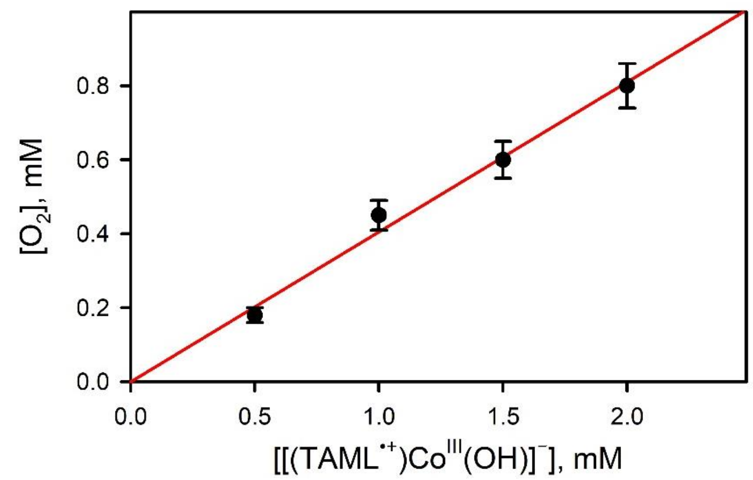

Figure S10. Plot of concentrations of **1** vs. concentrations of O_2 produced in the oxidation of H_2O_2 (0.50 equiv.) by **1** (0.50 – 2.0 mM) in Ar-saturated acetone at 25 °C. Intermediate **1** was generated by one electron oxidation of Li[Co(TAML)]·3H_2O (0.50 – 2.0 mM) with CAN (1.0 equiv.) in Ar-saturated acetone at 25 °C.

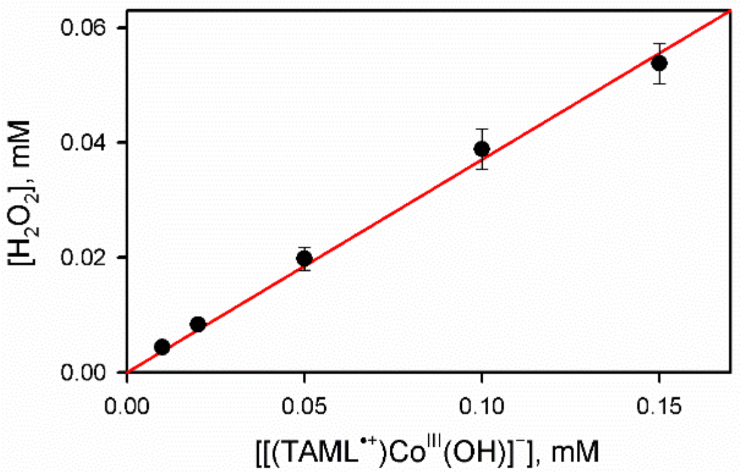

Figure S11. Plot of the concentration of **1** vs the concentration of H_2O_2 produced in the water oxidation reaction by **1** in the presence of H_2O (15 M) in Ar-saturated acetone at 25 °C. **1** was generated by oxidizing Li[Co(TAML)]•3H_2O (0.010 – 0.15 mM) with CAN (1.0 equiv) in Ar-saturated acetone at 25 °C.

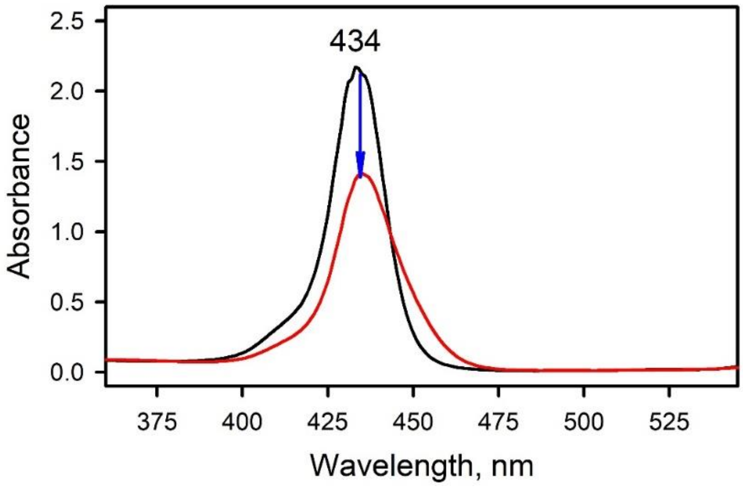

Figure S12. Absorption spectra of Ti-TPyP complex in the absence (black line) and presence (red line) of H_2O_2 formed during the water oxidation reaction catalyzed by **1** (0.020 mM) with H_2O (15 M) in Ar-saturated acetone at 25 ^oC. H_2O_2 was detected using the Ti-TPyP complex, which forms a titanium-peroxo species with a characteristic absorption maximum at λ_{max} = 434 nm. Intermediate **1** was generated by oxidizing Li[Co^{III}(TAML)]·3H_2O (0.020 mM) with CAN (1.0 equiv.) in Ar-saturated acetone at 25 ^oC.

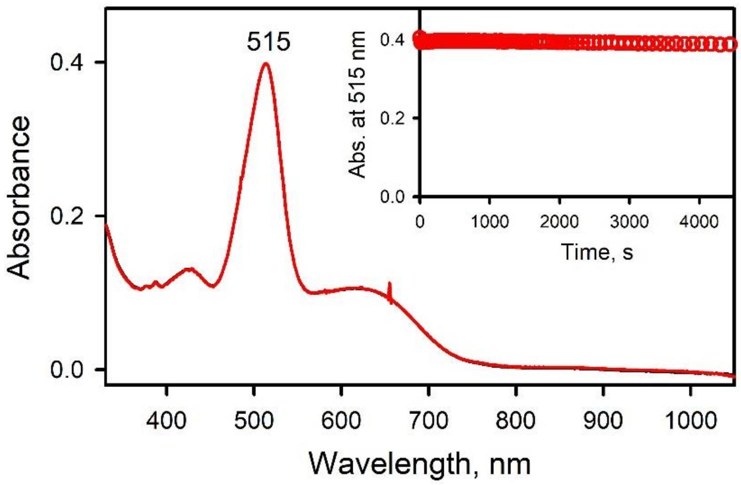

Figure S13. UV-vis spectra of Li[Co^{III}(TAML)]·3H_2O (0.10 mM) taken before (black line) and after (red line) the addition of H_2O_2 (1.0 mM) in Ar-saturated acetone at 25 ^{o}C. Inset shows time trace monitored at 515 nm due to Li[Co^{III}(TAML)]·3H_2O.

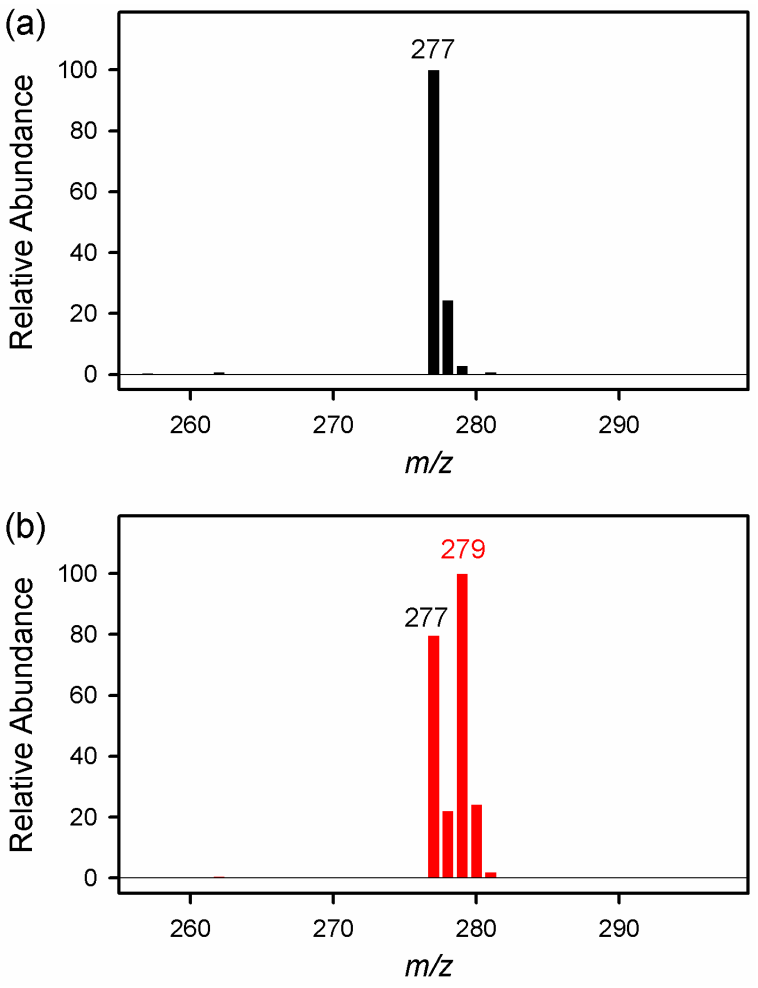

Figure S14. GC-MS spectra of the reaction solutions of the triphenylphosphine (PPh_3) oxidation to produce triphenylphosphine oxide ($O{=}PPh_3$) by (a) $H_2{}^{16}O_2$ and (b) $H_2{}^{18}O_2$, which were produced in the water oxidation reaction by **1** (0.10 mM) in the presence of water (15 M) in Ar-saturated acetone at 25 °C. The percentage of ^{18}O-labelled in $^{18}O{=}PPh_3$ (64%) was determined by comparing the relative abundances of the peaks at *m/z* = 277 and 279. Intermediate **1** was generated by one-electron oxidation of Li[Co^{III}(TAML)]·$3H_2O$ (0.10 mM) with CAN (1.0 equiv.) in Ar-saturated acetone at 25 °C.

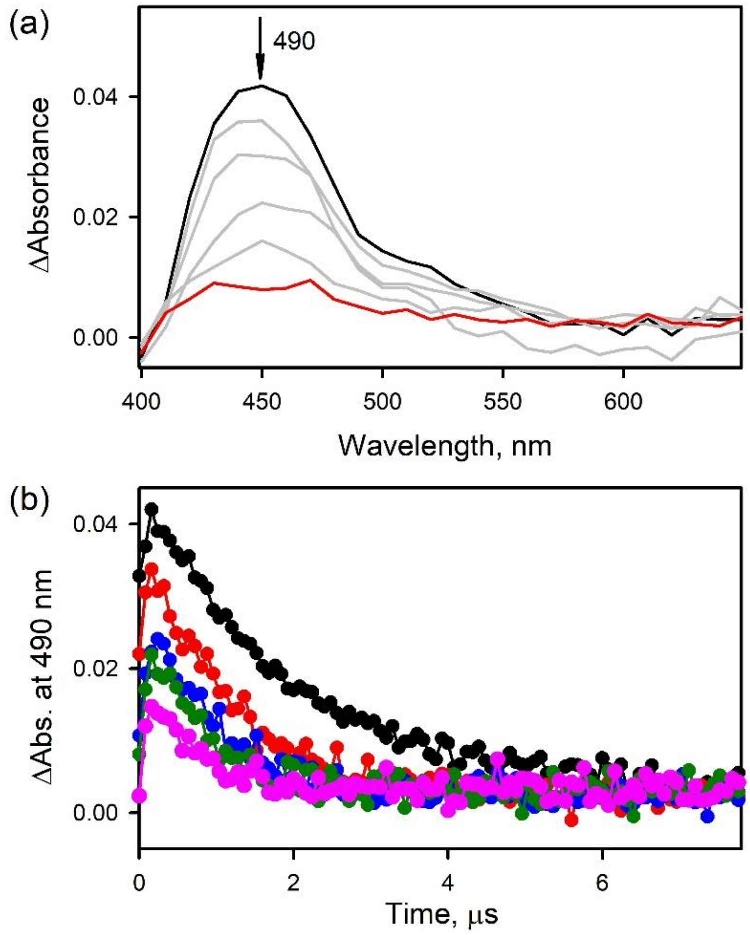

Figure S15. (a) Transient absorption spectral changes observed upon photoexcitation of Acr^{+}-Mes (0.20 mM) at λ_{ex} = 355 nm in Ar-saturated acetone at 25 ^{o}C (black line, 1.0 μs and red line, 100 μs). (b) Decay time profiles of $Mes^{•+}$ moiety of $^{3}(Acr^{•}–Mes^{•+})$ at 490 nm recorded in the presence of various concentrations of $Li[Co^{III}(TAML)]·3H_2O$ [black (0 mM), red (0.050 mM), blue (0.10 mM), green (0.15 mM), and pink (0.20 mM)]. The measurements were conducted after photoexcitation (λ_{ex} = 355 nm) of an Ar-saturated acetone solution of Acr^{+}–Mes (0.20 mM) at 25 °C.

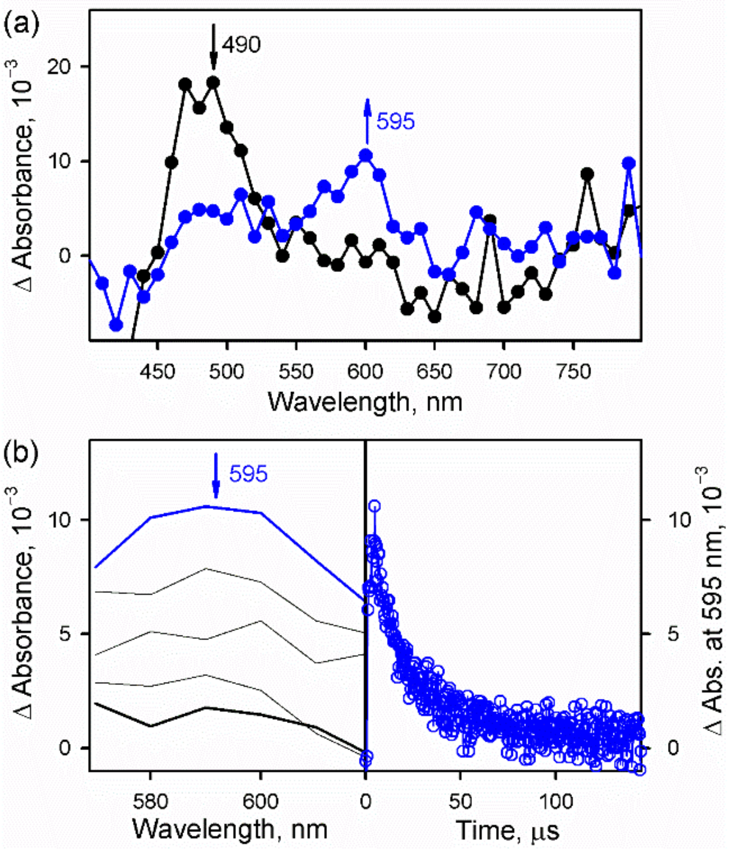

Figure S16. (a) Transient absorption spectral changes for the generation of **1** (blue circles) after photoexcitation (λ_{ex} = 355 nm) of an Ar-saturated acetone solution containing Li[Co(TAML)]•3H_2O (0.01 mM) and Acr^+-Mes (0.20 mM) (black circles) in the presence of H_2O (0.50 M) at 25 °C. (b) Transient absorption spectral changes (left panel) and the decay time profile (right panel) associated with the decay of **1** in the presence of Br_2Fc (1.0 mM) in Ar-saturated acetone at 25 °C. The spectral change was monitored at 595 nm.

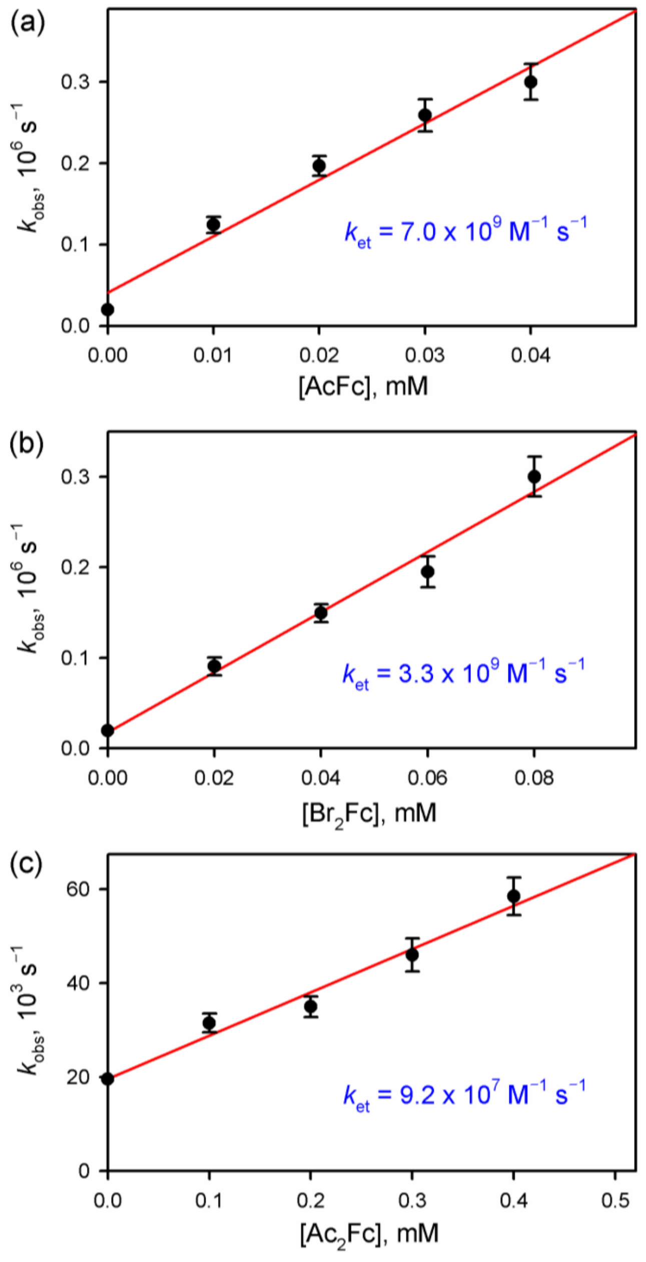

Figure S17. Plots of the pseudo-first-order rate constants (k_{obs}) of **1** at 595 nm vs. concentrations of electron donors (a) AcFc, (b) Br_2Fc, and (c) Ac_2Fc to determine the second-rate constant (k_{et}) in Ar-saturated acetone at 25 °C. Fast ET from electron donors (AcFc, Br_2FC and Ac_2Fc) to **1** was investigated using nanosecond laser-induced transient absorption measurements at 25 °C. **1** was generated in situ by ET from Li[Co(TAML)]•3H_2O (0.010 mM) to the triplet ET state of 9-mesityl-10-methylacridinium ion (^{3}Acr$^•$-Mes$^{•+}$, 0.20 mM) in Ar-saturated acetone at 25 °C.

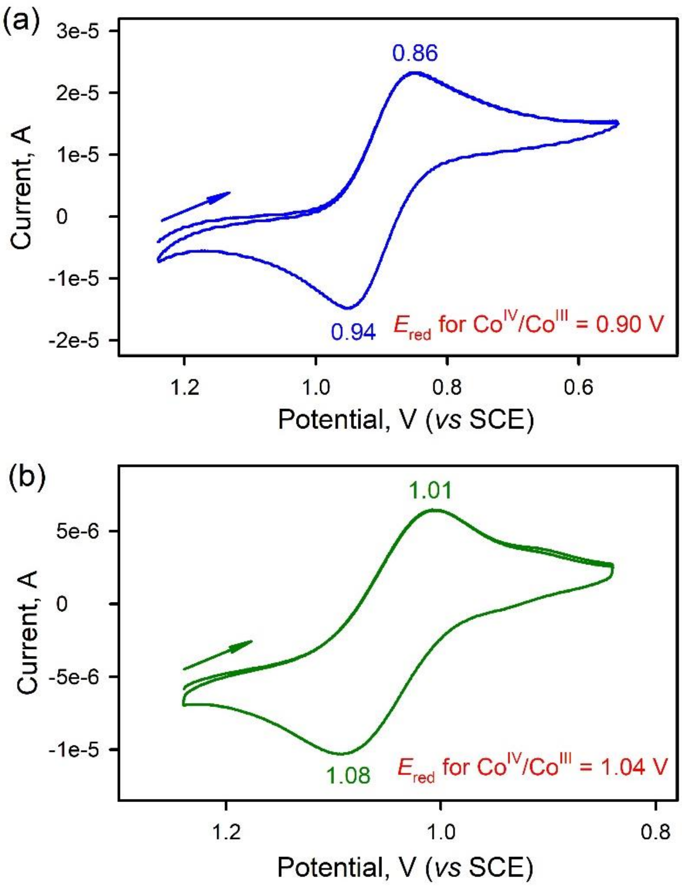

Figure S18. Cyclic voltammograms of (a) **1** (2.0 mM) and (b) **2** (2.0 mM) in Ar-saturated acetone containing $TBAPF_6$ (0.10 M) with a Pt working electrode at 0 °C and –40 °C, respectively. The scan rate was 100 mV s^{-1}. The measured potentials were recorded with respect to a reference electrode made from $Ag/AgNO_3$. All potentials were converted to the values of SCE by adding 0.29 V. All electrochemical measurements were carried out under Ar atmosphere. Intermediates **1** and **2** were generated upon addition of PhIO (3.0 equiv.) and HOTf (5.0 equiv.) to Ar-saturated acetone solution of Li[Co^{III}(TAML)]·$3H_2O$ (2.0 mM) at 0 °C and –40 °C, respectively.

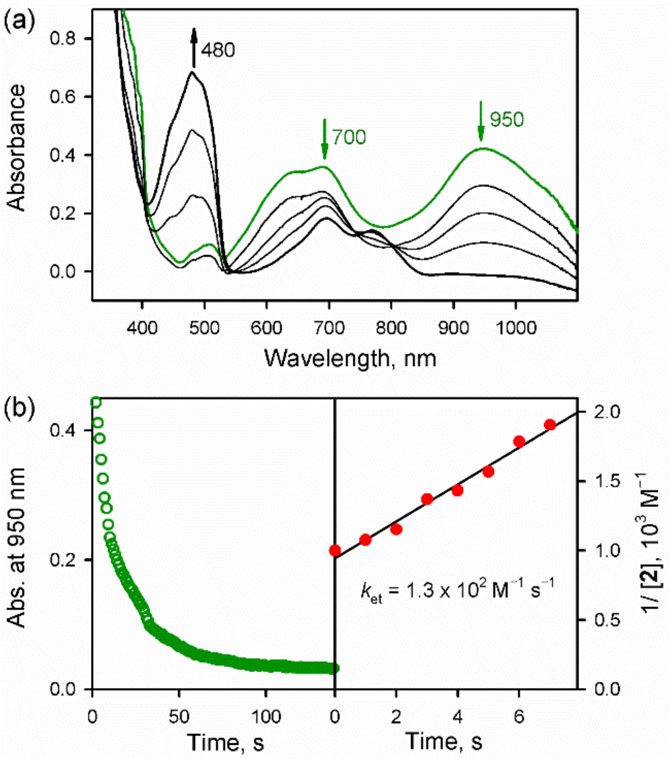

Figure S19. (a) UV-vis spectral changes observed in the ET reaction of **2** (1.0 mM, green line) and AcFc (1.0 mM) in Ar-saturated acetone at –80 °C. (b) Time course of absorbance change monitored at 950 nm due to the decay of **2** (left panel) and second-order plot of 1/[**2**] against time for ET from AcFc to **2** (1.0 mM) in Ar-saturated acetone at –80 °C (right panel).

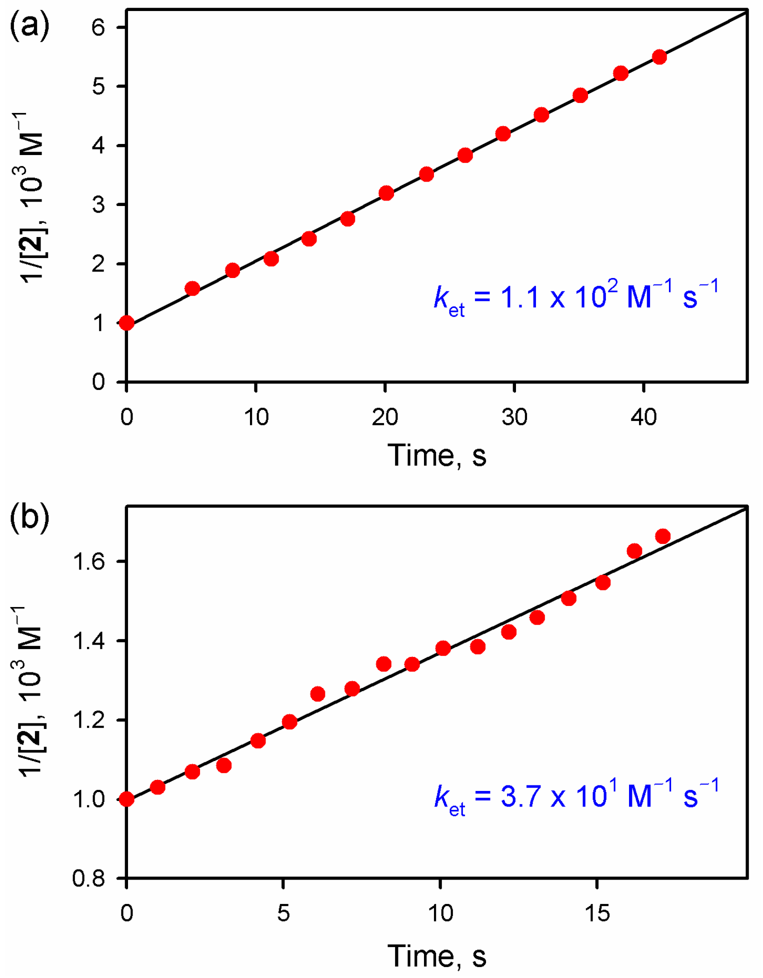

Figure S20. Second-order plots of 1/[**2**] against time in ET from (a) Br_2Fc and (b) Ac_2Fc to **2** in Ar-saturated acetone at –80 °C. Intermediate **2** was generated by reacting Li[Co^{III}(TAML)]·3H_2O (1.0 mM) with PhIO (3.0 equiv.) and HOTf (5.0 equiv.) and then reacted with electron donors (Br_2FC and Ac_2Fc) in Ar-saturated acetone at –80 ^oC.

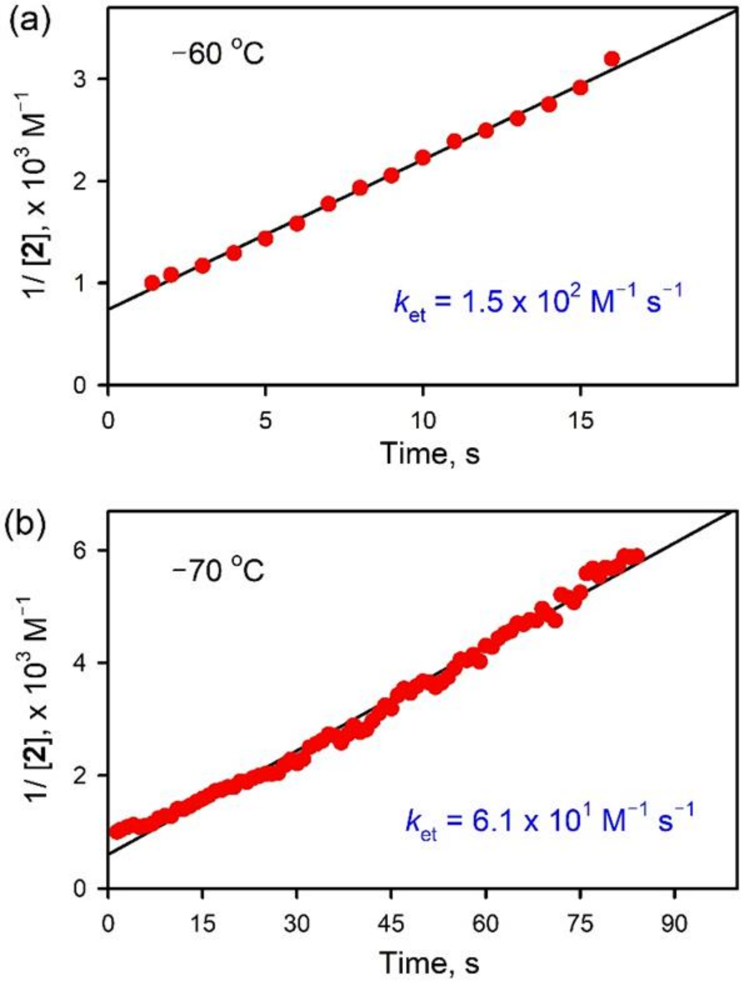

Figure S21. Second-order plots of 1/[**2**] against time in ET from Ac_2Fc to **2** in Ar-saturated acetone at (a) –60 and (b) –70 °C. Intermediate **2** was generated by reacting $Li[Co^{III}(TAML)]\cdot 3H_2O$ (1.0 mM) with PhIO (3.0 equiv.) and HOTf (5.0 equiv.) and then reacted with Ac_2Fc in Ar-saturated acetone.

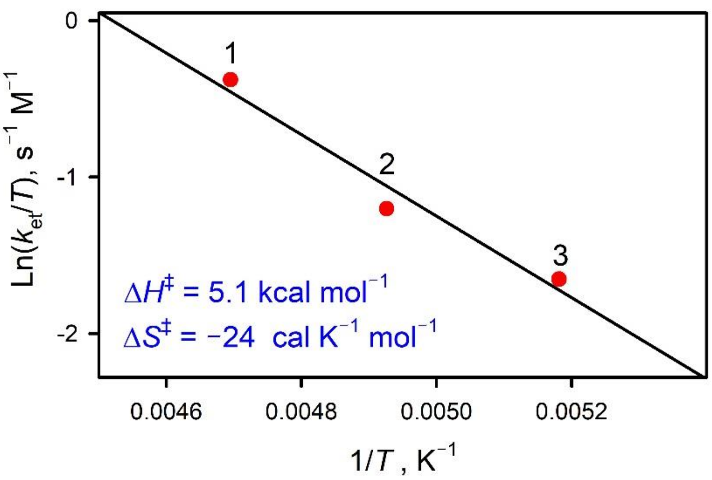

Figure S22. Eyring plot of the ET rate constant (k_{et}) from Ac_2Fc to **2** in acetone at various temperatures [–60, –70 and –80 ºC].

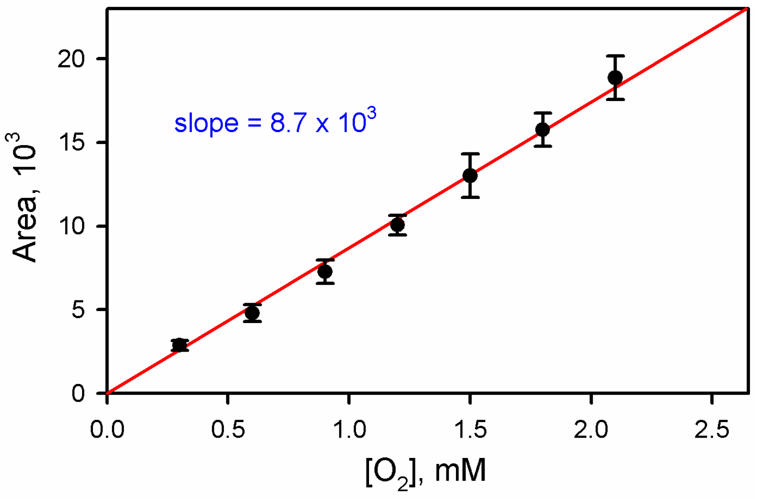

Figure S23. Calibration plot of the area of O_2 vs. the concentration of O_2 in Ar-saturated acetone. O_2 calibration was performed using GC by adding known concentrations of O_2-saturated acetone to Ar-saturated acetone at 25 °C.

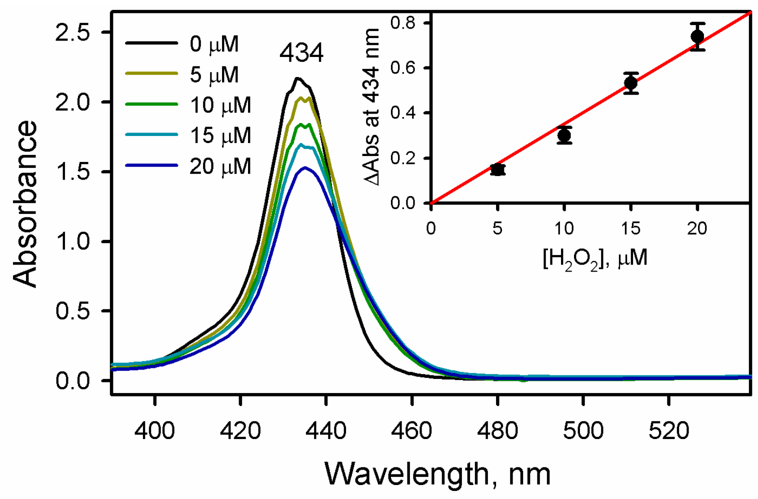

Figure S24. Absorption spectra of Ti-TPyP with authentic H_2O_2 (0 to 20 μM) in Ar-saturated acetone at 25 °C. The inset shows a plot of ΔA_{434} nm vs. the concentration of H_2O_2, providing a calibration curve. The concentration of H_2O_2 in the sample solution was determined by comparing its absorbance change at 434 nm (ΔA_{434} nm) to this standard calibration curve.
